# Intracellular Protein S-Nitrosylation—A Cells Response to Extracellular S100B and RAGE Receptor

**DOI:** 10.3390/biom12050613

**Published:** 2022-04-20

**Authors:** Monika Zaręba-Kozioł, Michał Burdukiewicz, Aleksandra Wysłouch-Cieszyńska

**Affiliations:** 1 Mass Spectrometry Laboratory, Institute of Biochemistry and Biophysics, Polish Academy of Sciences, Pawińskiego 5a, 02-106 Warsaw, Poland; m.zareba-koziol@nencki.edu.pl; 2 Laboratory of Cell Biophysics, Nencki Institute of Experimental Biology, Polish Academy of Science, Pasteura 3, 02-093 Warsaw, Poland; 3 Clinical Research Centre, Medical University of Białystok, Kilińskiego 1, 15-369 Białystok, Poland; michalburdukiewicz@gmail.com

**Keywords:** extracellular S100B, receptor RAGE, S-nitrosome, SNOSID, mass spectrometry

## Abstract

Human S100B is a small, multifunctional protein. Its activity, inside and outside cells, contributes to the biology of the brain, muscle, skin, and adipocyte tissues. Overexpression of S100B occurs in Down Syndrome, Alzheimer’s disease, Creutzfeldt–Jakob disease, schizophrenia, multiple sclerosis, brain tumors, epilepsy, melanoma, myocardial infarction, muscle disorders, and sarcopenia. Modulating the activities of S100B, related to human diseases, without disturbing its physiological functions, is vital for drug and therapy design. This work focuses on the extracellular activity of S100B and one of its receptors, the Receptor for Advanced Glycation End products (RAGE). The functional outcome of extracellular S100B, partially, depends on the activation of intracellular signaling pathways. Here, we used Biotin Switch Technique enrichment and mass-spectrometry-based proteomics to show that the appearance of the S100B protein in the extracellular milieu of the mammalian Chinese Hamster Ovary (CHO) cells, and expression of the membrane-bound RAGE receptor, lead to changes in the intracellular S-nitrosylation of, at least, more than a hundred proteins. Treatment of the wild-type CHO cells with nanomolar or micromolar concentrations of extracellular S100B modulates the sets of S-nitrosylation targets inside cells. The cellular S-nitrosome is tuned differently, depending on the presence or absence of stable RAGE receptor expression. The presented results are a proof-of-concept study, suggesting that S-nitrosylation, like other post-translational modifications, should be considered in future research, and in developing tailored therapies for S100B and RAGE receptor-related diseases.

## 1. Introduction

S100B is a small, EF-hand-type, calcium-binding protein, expressed by various vertebrates’ cells. It is a multifunctional protein [1,2,3]. S100B contributes to humans’ physiological processes, i.e., brain and muscle development and regeneration and adipose tissue innervation. Its overexpression correlates to multiple diseases. High levels of S100B are characteristic of Down Syndrome, Alzheimer’s disease, Creutzfeldt–Jakob disease, schizophrenia, multiple sclerosis, brain tumors, epilepsy, melanoma, myocardial infarction, muscle disorders, and sarcopenia [4,5,6].

Inhibiting S100B’s activities related to human diseases, without disturbing the protein’s physiological functions, is a vital goal of drug and therapy design [7,8,9].

S100B is an extracellular signaling factor [1]. It is a Damage-Associated Molecular Pattern (DAMP) molecule, released to the extracellular milieu by damaged tissues. There are also reports that S100B is actively released, i.e., by astrocytes, enteric glia or adipocytes, in a regulated manner. However, the detailed mechanism of how the cytoplasmic S100B is secreted to serve functions outside the cells is not yet fully understood [10,11,12,13]. The protein does not have an n-terminal or other signal peptide, required by known secretion systems. A non-classical S100B secretion by exosomes has been proposed, involving AHNAK/S100A10/annexin2-dependent exocytosis [14]. A recent study suggested the role of calsyntenin-3β in regulating the release of the protein in adipose tissue [15].

Extracellular S100B (exS100B) interacts with several receptors on the cell’s surface, inducing intracellular responses [16,17]. This study focused on the consequences of exS100B interactions with the Receptor for Advanced Glycation End-products (RAGE) [18].

RAGE is a multiligand, immunoglobulin-like receptor, existing in vivo as several isoforms of different sequence lengths [19]. Most human cells express a full-length transmembrane RAGE receptor during healthy development [20], but only a few types, in the lungs and skin, produce the protein after development completion [21,22]. However, in adult organisms, high RAGE expression is triggered at the surface of non-developing cells by the appearance of the receptor’s extracellular ligands, particularly in the presence of exS100B protein [6,23].

The biological outcome of exS100B activity depends on the stimulated cell type and the protein’s local concentration. Nanomolar physiological concentrations of exS100B are usually beneficial and, for example, favor Ca^2+^ homeostasis, cell differentiation, and tissue repair [6,12]. High, micromolar exS100B concentration often correlates with destructive processes: inflammation, melanoma progression, neurodegenerative diseases, neuroinflammation, aging, heart infarction, and interferes with tissue repair [6,12,24,25]. Low levels of exS100B are neurotrophic; highly increased exS100B causes neuronal death, but both functions, at least in part, depend on RAGE receptor activation [2,3,21,26].

In S100B-induced microglia migration, the concentration of exS100B determines which RAGE-dependent posttranslational phosphorylation pathways are activated [23]. Treatment of astrocytes with a low concentration of exS100B stimulates a subtle proinflammatory reaction, promoting glial scars. However, micromolar exS100B levels turn astrocytes into a neurodegenerative phenotype. In intestinal inflammation and celiac disease, exS100B, released in large amounts by enteric glial cells (EGCs), leads to RAGE overactivation [27]. Productive muscle regeneration depends on a scheme of time-dependent changes in the level of exS100B released by injured muscles [28].

Previous studies proposed that a particular functional outcome of exS100B, at least partially, depends on activating different intracellular signaling pathways, regulated by posttranslational protein phosphorylation [29,30]. Frequently linked with exS100B functions, were the attenuation of reactive oxygen species (ROS) production by NADPH oxidase and changes in the expression and activity of enzymes responsible for the level and reactivity of nitric oxide (NO) [31,32,33].

ROS and NO are central players in the cellular response to biological signals, based on redox modifications of specific cysteine sites in proteins. Reversible protein S-nitrosylation (SNO) is a crucial signaling post-translational modification PTM [34,35]. Alongside other posttranslational modifications, networks of proteins, with selectively S-nitrosylated thiols—the S-nitrosomes—form a specific adaptive system that responds to intra- and extracellular stimuli. S-nitrosome homeostasis is essential in human physiology [36]. Its disruption is a fundamental mechanism in many pathologies in which exS100B plays a role, e.g., neurodegenerative disorders, cardiac disease, muscle disease, inflammation, and cancer [37,38,39,40,41,42]. From the importance of ROS/NO signaling in some of the illnesses, emerged new targeted methods of treating them, with compounds focused on the action and production of ROS/NO [43,44].

In this work, we tested a hypothesis that intracellular protein S-nitrosylation is one of the tools used by cells to achieve the different effects of exS100B, and whether the RAGE receptor plays a role in these processes.

S-nitrosylation of a protein in a specific biological state is an effect of complicated crosstalk of multiple factors. Among them are the levels of local NOS activity and NO production, the activity of protein transnitrosylases and denitrosylases (similar to enzymes regulating other posttranslational modifications of proteins, i.e., phosphorylation), the presence of metal ions, and reactivity of many low-molecular-weight compounds, including reactive oxygen species and glutathione [45]. The most reliable way is to determine S-nitrosylated proteins in a specific biological environment, experimentally. To do this, we used an established mass spectrometry-based identification of S-nitrosylated proteins, specifically enriched using a Biotin Switch Technique [46]. Wild-type Chinese hamster ovary (CHOWT) cells were a simple model system. The CHO cells are among the best characterized, easy-to-manipulate mammalian cells, often used to produce recombinant, post-translationally-modified human proteins. The sequence similarity of Chinese hamster and human proteins is 95.65% for S100B, and more than 90% for the membrane-bound RAGE receptor. Unstimulated CHOWT cells do not express RAGE. Stable human RAGE expression on the surface of CHO cells was established by transfecting the cells with an expression vector, under an myeloproliferative sarcoma virus (MPSV) long terminal repeat (LTR) promoter, with subcloned human RAGE cDNA. CHOWT, together with CHO cells with stable overexpression of full-length human RAGE receptor (RAGECHO), served as a cellular model for studying some intracellular signaling mechanisms, induced by RAGE activation with ligands [47].

A crucial basis for our study design was the remarkable proteolytic stability of the extracellular S100B protein, well validated in studies on the use of the protein as a diagnostic marker [48].

With this study, we propose that analysis of intracellular S-nitrosylation and other PTMs may be essential to better understand the pertinent signaling pathways in developing tailored therapies for S100B and RAGE receptor-related diseases.

## 2. Materials and Methods

### 2.1. Expression and Purification of Recombinant S100B Protein

The synthetic gene encoding for bovine S100B was cloned into pAED4 plasmid and expressed in Escherichia coli utilizing the T7, AmpR expression system. Bacterial cells (HMS174 (DE3)) were grown in LB medium at 37 °C. Expression was induced by the addition of 0.4 mM isopropyl 1-thio-β-d-galactopyranoside (Sigma-Aldrich) at A600 = 0.8, and the bacterial culture was raised for two hours. The overexpressed protein was purified as described previously [49]. Analytical reverse phase HPLC and mass spectrometry confirmed the absence of any fragmented or modified protein. Protein concentration was estimated from its absorbance at 280 nm. The molar extinction coefficient for S100B protein (ϵmolar = 10.026) was determined experimentally by measuring the UV signal at 280 nm for a standard S100B solution. The standard S100B sample concentration was established by amino acid analysis at BioCentrum Ltd. (Kraków, Poland). Namely, the protein samples were hydrolyzed in the gas phase using 6 M HCl at 115 °C for 24 h. The free amino acids were converted into phenylthiocarbamyl derivatives and analyzed by high-pressure liquid chromatography (HPLC) on a PicoTag 3.9 × 150-mm column (Waters, Milford, MA, USA).

### 2.2. Cell Culture

CHO WT and full RAGE transfected CHO (CHORAGE) cells were a kind gift from Dr. Angelica Bierhaus from Heidelberg University. The cells were cultured at 37 °C in a humidified atmosphere containing 5% CO_2_ in DMEM medium (Gibco, Big Cabin, OK, USA) supplemented with 10% fetal bovine serum (FBS), 100 U/mL penicillin (Gibco, Big Cabin, OK, USA), and 100 U/mL streptomycin (Gibco, Big Cabin, OK, USA). Twenty-four hours before S100B treatment, the intact cells were washed with PBS and cultured in DMEM medium supplemented with 1% FBS.

### 2.3. Cell Cytotoxicity Assay (MTT)

MTT (3-[4,5-dimethylthiazol-2-yl]-2,5-diphenyl tetrazolium bromide) assay is a useful colorimetric test for detection of cell viability. Cells were starved in DMEM medium with 1% FBS for 24 h. Then, various concentrations of S100B were added for different time intervals as indicated, and cell viability was determined by the MTT assay as described previously [50]. The percentage of cell viability was calculated using the equation: (mean OD of treated cells/mean OD of control cells) × 100. Each experiment was performed at least three times, with five replicates for each condition.

### 2.4. Cell Line Treatment

The confluent CHOWT and CHORAGE cell lines, after 24 h starving, were incubated for 24 h with different concentrations of S100B protein (10 nM, 100 nM, 500 nM, 1.0 μM, 2.0 μM). After this time, confluent cells were scraped and resuspended in 250 mM HEPES buffer, pH 7.7 containing 1.0 mM EDTA and 0.1 mM neocuproine. Cells were homogenized using a Potter glass homogenizer (Sigma-Aldrich, St. Louis, MO, USA).

### 2.5. Western Blot Analysis of iNOS and RAGE Protein Expression

For the next phase, 20 µg of protein cell extracts were separated using a reducing 12% SDS–PAGE and transferred onto PVDF membrane. The membranes were blocked with casein-based buffer (Sigma-Aldrich, St. Louis, MO, USA), incubated with primary antibodies to iNOS and RAGE proteins, and then probed with secondary antibodies raised against the appropriate species. Loading of identical amounts of protein fractions on each line was confirmed using antibodies against GAPDH protein and by Ponceau S staining of the blots. Proteins were detected using ECL chemiluminescence. The blots were scanned and quantified densitometrically. The relative protein abundances of iNOS and RAGE proteins were normalized to the level of GAPDH protein changes in expression were assessed with a heteroscedastic two-tailed t-test.

### 2.6. Biotin Switch Technique

Substitution of S-nitrosylated Cys (SNO-Cys) sites with S-biotinylated Cys in synaptosomal protein lysates was based on a previously described BST procedure (51). In our study, we optimized the concentration of used reagents and the time reactions to increase the specificity and sensitivity of the method. Protein fractions were dissolved in HEN buffer containing 250 mM Hepes pH 7.7, 1 mM EDTA, and 0.1 mM neocuproine. To avoid rearrangements of the cysteine thiol modifying groups, the protein fractions were treated with a blocking buffer solution containing 250 mM HEPES pH 7.7 1 mM EDTA and 0.1 mM neocuproine 5% SDS, 20 mM MMTS at 50 °C for 20 min with agitation. Protein extracts were precipitated with acetone to remove an excess of reagents and resuspended in the same volume of HEN buffer with 2.5% SDS. The obtained protein solutions were divided in half. One part was treated with a mixture of 400 µM Biotin-HPDP and 5.0 mM sodium ascorbate. The second half was used as a negative control for experiments and treated with 400 µM biotin-HPDP without sodium ascorbate. All samples were incubated in the dark for 1.5 h at room temperature (RT). Samples from the Biotin Switch assay were diluted with two volumes of neutralization buffer (20 mM Hepes, pH 7.7, 100 mM NaCl, 1 mM EDTA) and 100 μL neutravidin–agarose was added and incubated for 1 h at room temperature with agitation. Beads were washed five times with wash buffer (20 mM Hepes, pH 7.7, 600 mM NaCl, 1 mM EDTA) and incubated with elution buffer (50 mM TRIS, pH 8.0, 1 mM EDTA, and 50 mM DTT) for 20 min at room temperature with gentle agitation. Eluted proteins were separated in 12% SDS–PAGE gel and afterward transferred to PVDF membranes (0.22 μm). PVDF membranes were blocked with nonfat dried milk and incubated with biotin-recognizing antibodies. Protein bands were detected using ECL-chemiluminescence system (Amersham, London, UK).

### 2.7. Western Blot Detection of Differential S-Nitrosylation in the Cell Lysates after Treatment with S100B Protein

Cell lysates (total and enriched SNO fractions) were separated using 12% SDS-PAGE and transferred to PVDF membranes (0.22 μm). The membranes were blocked with casein-based buffer (Sigma-Aldrich, St. Louis, MO, USA), incubated with primary antibodies specific for a protein of interest followed by secondary HRP-conjugated antibodies. Protein bands were detected using the ECL chemiluminescence system (Amersham, London, UK). GelQuant software (Version 2.7, Neve Yamin, Israel) compared densitometrically the amounts of S-nitrosylated proteins under all studied conditions. A heteroscedastic two-tailed t-test was used to assess the changes in S-nitrosylation statistically.

### 2.8. SNOSID Procedure

According to the previously described Biotin Switch procedure, the S-nitrosylated cysteines in proteins from cell lysates were selectively exchanged to S-S-biotinylated cysteines. However, the cell lysates after biotinylation were digested to by peptides using sequencing grade modified trypsin (Promega V 5111, Madison, WI, USA) for 16 h at 37 °C. The digestion was terminated using a cocktail of protease inhibitors. The mixture of tryptic peptides was incubated with 100 μL of neutravidin beads at room temperature for 1 h, and the neutravidin beads were washed five times in a 1 mL of wash buffer. Neutravidin-bound peptides were eluted with 150 μL of elution buffer and concentrated in a SpeedVac. Trifluoroacetic acid was added to the peptide solution to achieve a final concentration of 0.1%. nLC-MS and nLC-MS/MS analyzed samples.

### 2.9. LC-MS and LC-MS/MS Analysis

For each enriched SNO-peptides containing a sample, separate LC-MS (profile type, peak amplitude data) and LC-MS/MS (peptide identification data) runs were performed. Nano Aquity Liquid Chromatography system (Waters, MA, USA) coupled with LTQ-FTICR mass spectrometer (Thermo Scientific, Waltham, MA, USA) was used.

SNO-peptides in 0.1% formic acid/water were loaded from a cooled (10 °C) autosampler tray to a pre-column (Symmetry C18, 180 µm × 20 mm, 5 µm Waters) and resolved on BEH130 column (C18, 75 mm × 250 mm, 1.7 mm, Waters, MA, USA) in a gradient of 5–30% acetonitrile/water for 70 min at 0.3 µL/min flow rate. UPLC system was directly connected to the ion source of the mass spectrometer. All MS runs were separated by blank ones to reduce the carry-over of peptides from previous samples. All MS runs were carried out in triplicate (for FVB and hAPP). The spectrometer’s resolution was set to 50,000 with the m/z measurement range of 300–2000. Raw LC-MS data were converted to a data format of NMRPipe software (http://spin.niddk.nih.gov/NMRPipe, accessed on 8 March 2022, Version Number 20210915, Manufacturer, Bethesda, MD, USA) by an in-house designed finnigan2Pipe data conversion tool [51,52]. MSparky (http://www.ire.pw.edu.pl/~trubel/soft/ms/msparky (accessed on 8 March 2022), (Version Number 2.3, Warsaw, Poland), an in-house modified version of Sparky NMR software (http://www.cgl.ucsf.edu/home/sparky (accessed on 8 March 2022)), was used to convert the LC-MS data into 2D heat maps where the peptide’s m/z is one dimension and its LC retention time is the second dimension. MSparky allows for interactive validation and inspection of the data. In the LC-MS/MS runs, up to ten fragmentation events were allowed for each parent ion. MascotDistiller software (version 2.2, Matrix-Science, Boston, MA, USA) was used to process the datasets of parent and daughter ions. Mascot search engine (version 2.3, Manufacturer, Boston, MA, USA) was used to survey data against UniProtKB/Swiss-Prot (version 51.5, The UniProt Consortium, Hinxton, UK) database. The Mascot search parameters were: taxonomy Mus musculus, fixed modification-cysteine carbamidomethylation, variable modification-methionine oxidation, peptide tolerance 40 ppm, fragment mass tolerance 0.8 Da, and as enzyme specificity was set to semi-trypsin. Lists of peptides’ sequences identified in all LC-MS/MS runs from FVB and hAPP synaptosomes were merged into one selected peptides list (SPL) by in-house developed software MascotScan (http://www.ire.pw.edu.pl/~trubel/soft/ms/mascotscan (accessed on 8 March 2022) (Warsaw, Poland). The SPL consists of sequences of peptides with Mascot scores >30 and unique m/z and LC retention time values. SPL list of sequences was used to label peaks in all 2D heat maps (profile data) with the same LC, m/z, and z coordinates with an in-house developed TagProfile [52]. The software allows for the correction of differences in peptide retention times due to changes in the quality of LC columns and slight differences in LC mobile phase content. Acceptance criteria included deviations in m/z value (20 ppm), retention time (10 min), envelope root mean squared error (i.e., a deviation between the expected isotopic envelope of the peak heights and their experimental values, 0.7), and charge state value following the SPL value. As a result, selected monoisotopic peaks on the 2D map were tagged with a peptide sequence. Peptides from the SPL list that were not automatically assigned by TagProfile to appropriate signals on 2D maps or those which did not meet all acceptance criteria were manually verified using the MSparky software.

The list of assigned MS sequences was simplified so that a single sequence corresponded to all peptides with parent entries which differed only by a charge state (z) value.

### 2.10. Functional Bioinformatic Analysis

Bioinformatics analyses were performed using Panther software (version 9.0; www.pantherdb.org (accessed on 8 March 2022), Celera Genomics, Foster City, CA, USA) and the STRING database. Gene Ontology and REACTOME proteins were classified based on biological process categories in GO annotations. Functional grouping was based on a Fisher Exact test *p* ≤ 0.05 and at least two counts. To identify functional annotations, associations, interactions, and networks within our dataset, STRING 9.1 (Search Tool for the Retrieval of Interacting Genes/Proteins, STRING CONSORTIUM, Lausanne, Switzerland) was used.

## 3. Results

### 3.1. Extracellular S100B Is Not Cytotoxic for CHOWT and RAGECHO Cells

**Figure 1:**
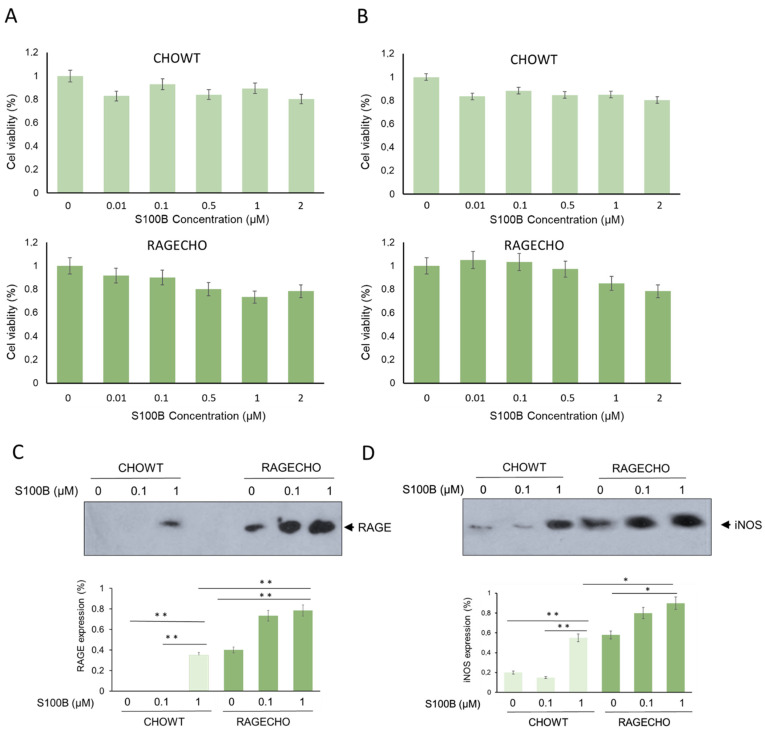
Cell viability, RAGE, and inducible nitric oxide synthase levels in CHOWT and RAGECHO cells—the model system to study changes in exS100B and RAGE-dependent intracellular S-nitrosylation. (**A**,**B**) The effects of 24 or 48 h treatment with exS100B on the CHOWT and RAGECHO cells’ viability. Cell viability measured using the methyl-thiazolyl diphenyltetrazolium bromide (MTT) method; (**C**) Western blot analysis of RAGE protein level after treatment of CHOWT and RAGECHO cells with different concentrations of exS100B. Blots are representative of three independent experiments. Bars show means  ±  SEM; * *p*  <  0.05; ** *p*  <  0.01; two-tailed *t*-test; (**D**) Western blot analysis and densitometric quantification of iNOS and RAGE protein levels after treatment of CHOWT and RAGECHO cells with different concentrations of exS100B. Blots are representative of three independent experiments. Bars show means  ±  SEM; * *p*  <  0.05; ** *p*  <  0.01; two-tailed *t*-test.

Using MTT-based cytotoxicity assay, we determined whether the chosen cell lines, CHOWT and RAGECHO, are viable in different extracellular S100B protein concentrations and can be used for differential proteomics studies. Cells were treated with increasing concentrations of S100B (0.01; 0.1; 0.5; 1; 2 µM) for 24 and 48 h. As shown in Figure 1A,B, the presence of extracellular S100B in the culture medium slightly affects the survival of cells, depending on concentration, time of incubation, and RAGE receptor presence. exS100B does not affect cell viability at low concentrations of 0.01 and 0.1 µM, and the cytotoxicity does not exceed 15% for higher (0.5–2 µM) concentrations. The viability of RAGECHO cells treated with high concentrations of S100B (0.5–2 µM) was slightly higher than for CHOWT cells. We used the 24-h treatment with low (0.1 µM) or high (1 µM) concentrations of S100B protein in all further experiments.

### 3.2. Extracellular S100B Modulates the Level of RAGE Receptor in CHO Cells

To better characterize the studied cell lines, we tested the expression level of RAGE receptors in CHOWT and RAGECHO cells, before and after treatment with exS100B protein, in low and high concentrations. We used western blot analysis and antibodies specific to RAGE to assess the protein level. As presented in Figure 1C, RAGE was undetectable in untreated CHOWT cells (lane 1) and observed in significant amounts in untreated RAGECHO cells (lane 4). Moreover, treatment with the high (lane 3), but not low (lane 2), exS100B concentrations induced RAGE expression in CHOWT cells, to a level similar to the unstimulated RAGECHO cells (*p* < 0.001). In RAGECHO cells, cultured in both low and high S100B amounts, the RAGE level doubled over the initial stable expression (*p* < 0.001; lanes 5 and 6), suggesting that under such conditions, exS100B activates the hamster’s RAGE expression. Western blots confirmed the expected absence of RAGE in untreated CHOWT cells and the steady level of RAGE receptors in untreated RAGECHO cells. They also indicated a specific, ligand-induced, ligand concentration-dependent RAGE expression pattern, upon incubation of CHOWT and RAGECHO with extracellular S100B.

### 3.3. RAGE Expression and Treatment with Extracellular S100B Modulate iNOS Protein Level in CHO Cells

We utilized antibodies specific to iNOS to determine if exS100B protein or membrane-bound RAGE modulates inducible nitric oxide synthase (iNOS) expression in CHO cells. Western blot (WB) analysis (Figure 1D) showed that the iNOS protein level was low in the control of untreated CHOWT cells, and the CHOWT cells induced by 0.1 µM S100B (lane 1 and 2). However, treatment of CHOWT cells with 1.0 µM S100B gave rise to a significant appearance of iNOS (*p* < 0.001; lane 3). In contrast, in the RAGECHO cells, high-level iNOS expression was observed, already, in the untreated cells (lane 4), and its amount increased to a similar level after treatment with 0.1 µM and 1.0 µM S100B protein (*p* < 0.001; lanes 5 and 6). Densitometric quantification of protein levels shows that iNOS and RAGE expressions change in the same direction.

### 3.4. RAGE Expression and Extracellular S100B Change the Patterns of Protein S-Nitrosylation in CHO Cells

The experiments described above confirmed that the model CHO cells are sensitive to the presence and concentration of exS100B protein. They also showed no significant cell death of S100B-treated cells that could disturb a comparative proteomic analysis.

**Figure 2:**
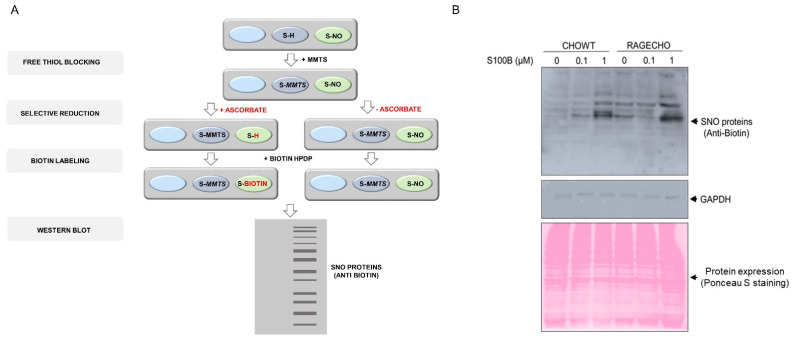
Protein S-nitrosylation in the CHOWT and RAGECHO cells (**A**). Schematic representation of protein S-nitrosylation analysis using the Biotin Switch Method; (**B**). Western blot analysis of protein S-nitrosylation in CHOWT and RAGECHO cells after treatment with 0.1 μM or 1.0 μM of extra-cellular S100B, and a whole-cell protein content in CHOWT and RAGECHO visualized by Ponceau S staining. Same intensities of GAPDH staining were the controls of sample loading.

Alterations in nitric oxide synthase activity and other cellular nitroso redox equilibrium elements impact the system of redox-dependent posttranslational modifications of reactive cysteines in proteins. After confirming that the RAGE receptor and extracellular S100B modulate iNOS protein levels, we checked if these factors influence S-nitrosylation of the intracellular protein cysteines. We confirmed no substantial changes in the overall cellular protein expression profile, under all studied conditions, for samples containing identical whole protein concentrations, using Ponceau S staining (Figure 2A). We then employed the Biotin Switch Technique (BST) to enrich the S-nitrosylated proteins. The method involves the selective substitution of labile S-NO groups by S-S-biotinylated derivatives (scheme on Figure 2A), later detecting marked proteins by western blot analysis with biotin-recognizing antibodies. Figure 2B presents the western blot results, revealing numerous protein bands across a broad mass range, derived by BST from identical amounts of protein fractions from untreated CHOWT and RAGECHO. The number and intensities of western blot bands were more elevated in RAGE-expressing, untreated cells, suggesting a higher overall basal protein S-nitrosylation. The bands changed after treating cells with exS100B, but changes were not proportional to the exS100B concentration. Most evident was the decreased intensity of S-S-biotinylated protein bands in RAGECHO cells treated with 0.1 µM exS100B.

### 3.5. Mass Spectrometry-Based Identification of Specific Protein S-Nitrosylation Sites in CHOWT and RAGECHO Cells Unstimulated and Treated with Extracellular S100B

**Figure 3:**
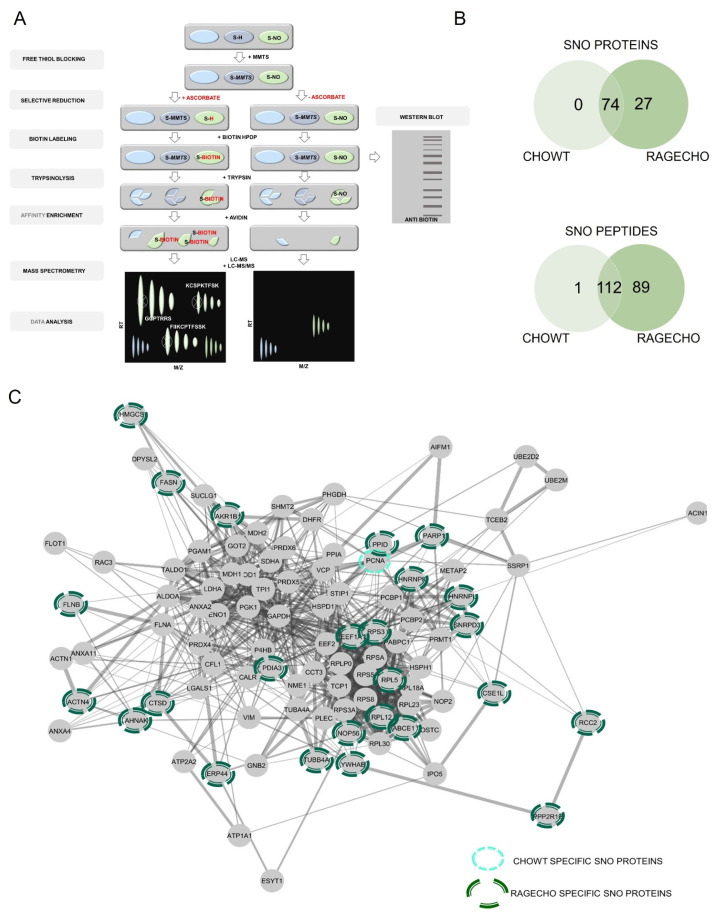
SNOSID analysis of basal protein S-nitrosylation in CHOWT and RAGECHO cells. (**A**) The experimental scheme of protein S-nitrosylation site analysis (SNOSID), (**B**) Venn diagrams comparing numbers of basal SNO proteins or SNO peptides identified for WTCHO and RAGECHO cells. (**A**) Schematic representation of a mass spectrometry-based approach for large-scale, site-specific S-nitrosylation analysis. Stable biotin derivatives substituted labile S-nitrosylation groups on cysteine thiols. After trypsin digestion, the biotinylated peptides were enriched on avidin resin and analyzed using MS in two independent runs (LC-MS and LC-MS/MS). MSparky allowed for site-specific data analysis; (**B**) Venn diagram comparisons of the numbers of SNO proteins and sites identified in CHOWT and RAGECHO cells; (**C**) String interaction network of all identified S-nitrosylated proteins. Light green marked are proteins, specifically SNO in CHOWT cells; dark green marked are proteins present only in the RAGECHO cell line.

We employed mass spectrometry-based protein identification to validate our biochemical findings and identify the protein targets of S-nitrosylation and S-nitrosylated cysteine sites, specific for CHOWT and RAGECHO, with and without exS100B. To do that, we used the SNO-site identification procedure (SNOSID), allowing for non-targeted proteomic identification of S-nitrosylated cysteine residues in protein fragments [53]. As Figure 3A presents, SNOSID relies on a selective exchange of labile SNO groups on protein cysteines to stable thiol derivatives of biotin, as in BST. The SNOSID procedure aims to detect particular S-nitrosylation sites, even in proteins with multiple cysteines in their sequence. For this, proteins, after derivatization of S-nitrosylated cysteines, were enzymatically digested with trypsin. Then, only the biotinylated tryptic fragments of the proteins attached to avidin beads. LC-MS-MS/MS identified the peptides released from the beads [44,45].

We designed our analysis to decipher the presence or absence of SNO protein targets and sites, as present or absent, under a given experimental condition. We did not compare quantitative SNO changes at this stage of our project. Furthermore, an S-nitrosylated peptide may be under-detected due to MS sensitivity drop-out effects. Thus, while a positive (presence) result is reliable, the absence of peptide identification should be interpreted with care.

Initially, we used SNOSID analysis to identify the basal S-nitrosome of unstimulated CHOWT and RAGECHO cells. The result was a list of 201 distinct peptide sequences, with at least one cysteine residue, assigned in our experiments as endogenously S-nitrosylated (Supplementary Table S1). The identified peptides are fragments of only 101 separate protein sequences. Eighty-nine peptides from twenty-seven proteins were detected, exclusively, in untreated RAGECHO cells. Only a single SNO peptide fragment of annexin A11 was unique in the CHOWT S-nitrosome (Figure 3B). The vast majority (99 of 101) of proteins with assigned SNO site, under basal conditions, contain more than one cysteine in their sequence. The contribution of identified S-nitrosylated residues, among all cysteines in a particular protein, differed significantly. For proteins with multiple cysteines, we detected a single S-nitrosylation site for forty-three of them, two sites for thirty-nine, three sites for sixteen, four sites for five, five sites for two, and as many as seven sites for one protein (Table S1). In some proteins, all of the multiple cysteines are S-nitrosylated, while in others, the modification is specific. Under experimental conditions, presented here, a good example is the apoptotic chromatin condensation inducer 1 (ACINUS1), in which solely one of the forty-three cysteines has been modified. Such results suggest that S-nitrosylation in the studied model cells is a selective posttranslational modification of protein cysteines.

We used the protein interactome database (STRING) for bioinformatic analysis of the experimental data. Figure 3C schematically shows the interactome of SNO proteins, identified by us, emphasizing the twenty-seven uniquely S-nitrosylated in RAGECHO and one in CHOWT cells. It revealed that the hundred-and-one identified S-nitrosylated proteins are a part of a well-defined interaction network.

### 3.6. Extracellular S100B Protein Tunes the Pattern of Intracellular Proteins S-Nitrosylation in CHOWT and RAGECHO Cells

**Figure 4:**
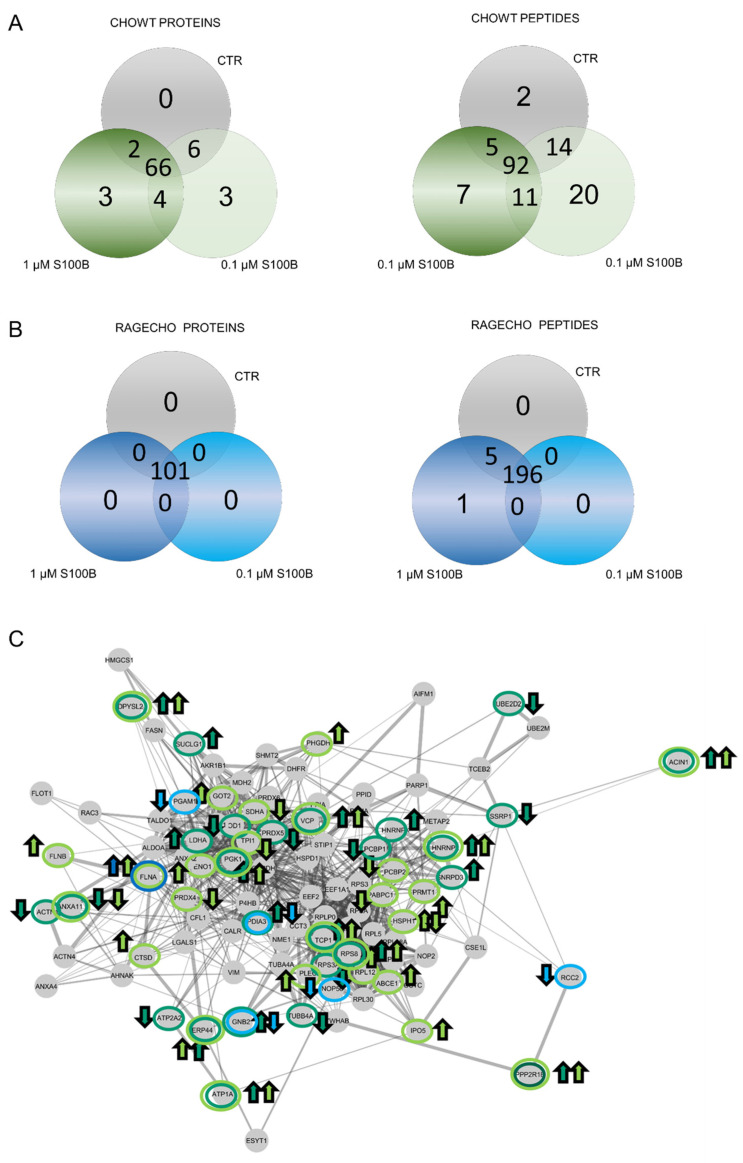
Impact of extracellular S100B treatment on the CHOWT and RAGECHO cells S-nitrosome. (**A**) Venn diagrams compare the numbers of SNO proteins identified in CHOWT after treatment with the low and high concentrations of S100B; (**B**) Venn diagrams compare the numbers of SNO proteins identified in CHOWT after treatment with low and high extracellular S100B; (**C**) String network analysis of all identified SNO proteins. Marked are S100B-induced SNO proteins in CHOWT and RAGECHO cells. Arrows reflects the direction of the change (upregulation and downregulation).

After identifying the basal S-nitrosome of unstimulated cells, we performed the SNOSID procedure for CHOWT cells treated with either 0.1 µM or 1.0 µM exS100B concentration. We used Venn diagrams to select numbers of S-nitrosylated proteins characteristic for a particular experimental setup and SNO proteins present under all conditions (Figure 4A,B). In CHOWT cells, we identified sixty-six stably S-nitrosylated proteins present under all experimental conditions. However, eighteen proteins from CHOWT cells were critically involved in the S100B-related signaling. We found three proteins, exclusively SNO, after treatment with 0.1 µM (cathepsin D, filamin B, and the ATP-binding cassette subfamily E member 1) and three exclusively SNO after treatment with 1.0 µM (small nuclear ribonucleoprotein Sm D3, heterogeneous nuclear ribonucleoprotein L, and protein disulfide-isomerase A3). Four SNO proteins were present in cells treated with either 0.1 µM or 1.0 µM exS100B and absent in the controls (Apoptotic chromatin condensation inducer in the nucleus, Protein Ppp2r1b, Heterogeneous nuclear ribonucleoprotein K, Endoplasmic reticulum resident protein 44); two (Succinate dehydrogenase [ubiquinone] flavoprotein subunit and mitochondrial Poly(rC)-binding protein 2) are present in control and 1.0 µM but not 0.1 µM of exS100B. Six proteins are in control and 0.1 µM but not 1.0 µM of S100B (Ubiquitin-conjugating enzyme E2 D2, Superoxide dismutase [Cu-Zn], FACT complex subunit SSRP1, 40S ribosomal protein S3a, Acetyl-Coenzyme A acetyltransferase 3, Sarcoplasmic/endoplasmic reticulum calcium ATPase 2). Table 1 collects the names of proteins that differentiate the specific experimental conditions, and Supplementary Table S2 presents a more detailed analysis of the biological functions of these proteins, using a current Gene Ontology database.

An analogous analysis at the level of individual SNO sites showed that ninety-two peptides were stably S-nitrosylated, in every condition, out of the two hundred and one (Figure 4A). Differential SNO sites are described in Table 2 and Supplementary Table S3. Only two Cys S-NO peptides are unique and characteristic only for untreated CHOWT cells. Twenty or seven Cys S-NO peptides are specific for CHOWT cells stimulated with 0.1 µM or 1.0 μM exS100B, respectively. Thirty more were present for both exS100B concentrations. The differential Cys S-NO peptides are fragments of 41 proteins. Thus, the number of proteins with differential SNO-sites is higher than the number of whole differential proteins (10 proteins). Such results suggest that multi-cysteine proteins S-nitrosylated at some site under one condition are possibly sequentially S-nitrosylated at different locations in another situation. Interactome analysis of S100B-dependent SNO proteins, using the STRING database of interacting proteins, showed a highly connected network, meaning that those proteins have more relations than expected for a random set of proteins of similar size, drawn from the genome (Figure 4C).

For RAGECHO cells, we identified all 101 SNO proteins in untreated cells and after their stimulation, with either low or high exS100B protein (Figure 4B). However, there were SNO sites in several proteins specific to a particular condition (Table 3, Supplementary Table S4). For example, treatment with 0.1 μM exS100B leads to denitrosylation of cysteines in peptides assigned to phosphoglycerate mutase 1, protein disulfide-isomerase A3, guanine nucleotide-binding protein G(I)/G(S)/G(T) subunit beta-2, nucleolar protein 56 and protein RCC2. Treatment with 1.0 μM exS100B induced an additional SNO site in filamin A.

In conclusion, our proteomic studies indicate that the appearance of the S100B protein outside the cell is a signal for changes in the intracellular S-nitrosome. The sets of S-nitrosylated proteins are different for low concentrations of exS100B, at which the WTCHO cells do not produce RAGE, and high concentrations of the ligand that induce RAGE expression. Incubation with exS100B of cells, constitutively expressing RAGE, causes only minor S-nitrosylation changes at a few new SNO sites.

### 3.7. Functional Categorization for Identified S-Nitrosomes—A Bioinformatic Analysis of Experimental Proteomic Data

The SNOSID/LC-MS-MS/MS proteomic study was consistent with the initial western blot observations, that exS100B and RAGE activity may tune the shape of S-nitrosome in CHO cells. Additionally, the SNOSID data identified individual S-nitrosylated proteins and cysteine residues, present commonly under all conditions, or differentially in the particular ones.

To better understand the biological roles of S-nitrosylation in CHOWT and RAGECHO cells, we searched for information on the identified SNO target proteins in a larger context of the human proteome. We conducted the NCBI Gene Ontology (GO) term enrichment analysis for identified SNO-proteomes, using the recent Panther software [54]. Unfortunately, though the genome database of CHO cells is available (http://www.chogenome.org (accessed on 1 July 2019), the Chinese hamster gene’s functional annotations are scarce. Thus, we used the NCBI Homologene program (http://www.ncbi.nlm.nih.gov/sites/homologene/ (accessed on 1 July 2019) for assigning human orthologues for the hamster proteins identified in our study. We used the identifiers of human orthologues as an input set for GO functional network analyses. Such analyses identified biological processes and pathways enriched in the analyzed experimental set, related to the background of the total human proteome.

**Figure 5:**
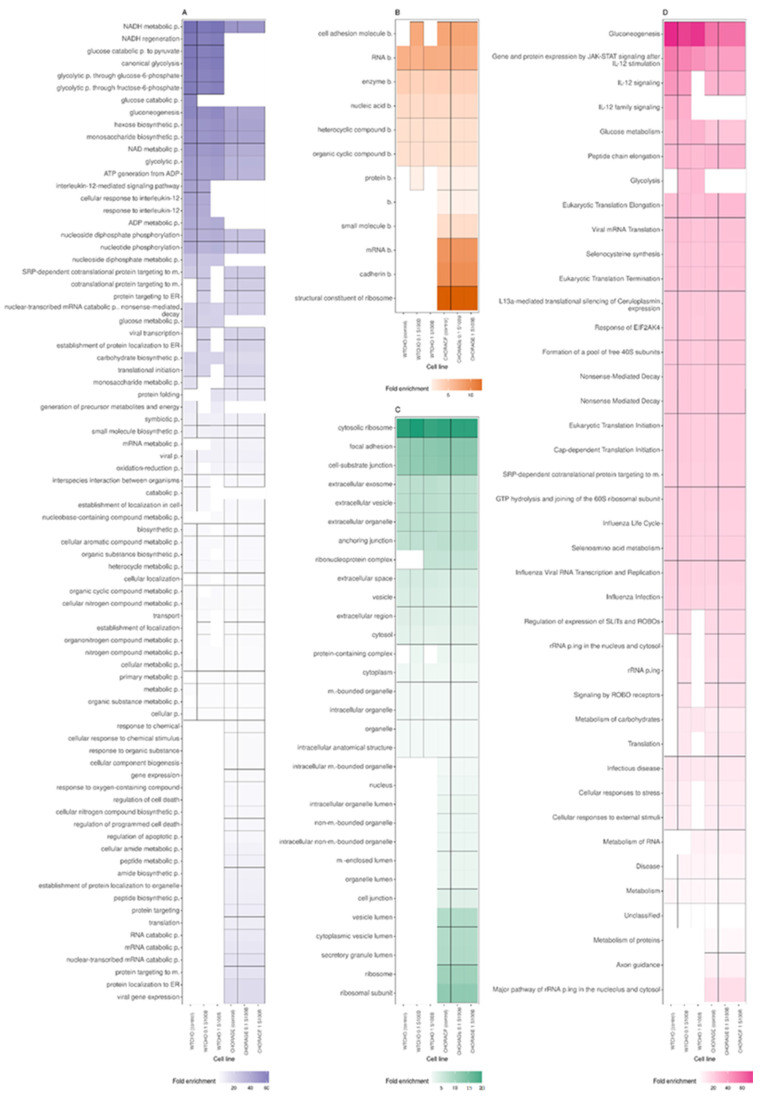
A comparison of Gene Ontology and KEGG Reactome analyses for S-nitrosylated protein sets detected for control CHOWT and RAGECHO cell cultures and the cells treated with 0.1 μM or 1.0 μM extracellular S100B protein. The analyzed categories are (**A**) GO Molecular Function, (**B**) KEGG (**C**) GO Cellular Component, (**D**) GO Biological Process.

Figure 5A,C,D compares the analyses in three GO domains: molecular function, biological process, and cellular component, performed separately for the six experimental variants of CHOWT and RAGECHO cell cultures.

Examination of the cellular component category showed that proteins affected by S-nitrosylation come from various cellular organelles, including cytosol, intracellular organelle lumen, ribosome, intracellular organelle, nucleus, and mitochondria (Figure 5C).

Interestingly, there are significant differences in the enriched terms between the CHOWT and RAGECHO cells. Compared to CHOWT, in RAGE-expressing cells, more S-nitrosylated proteins were detected from the nucleus, vesicle lumen, secretory granule lumen, and the ribosome attached to the endoplasmic reticulum. exS100B stimulation of CHOWT cells induces more SNO proteins, which are elements of the ribonucleoprotein and protein-containing complexes.

According to the GO molecular function category, a unique group of S-nitrosylation targets, namely proteins that are structural constituents of the ribosome and are involved in cadherin and rRNA binding (Figure 5A), are exclusively detected in the S-nitrosome of RAGECHO cells.

Significant differences are also in the overrepresentation of SNO proteins in the “biological process” category. For example, S-nitrosylated proteins belonging to the NADH regeneration, canonical glycolysis, gluconeogenesis, ADP metabolic process terms, are significantly enriched in CHOWT cells. Those from viral gene expression, protein localization to the endoplasmic reticulum (ER), protein targeting to the membrane, or RNA catabolic processes are found only in RAGECHO cells.

Treatment of cells with exS100B correlates with the S-nitrosylation of proteins involved in the interleukin 12 signaling pathway, targeting the ER, involved in viral transcription and protein folding. (Figure 5D).

According to KEGG analysis (Figure 5B), all S-nitrosylated proteins identified in this work assign to forty different Reactome terms. For twenty-three terms, there is a similar enrichment of SNO proteins, in cells expressing and not expressing RAGE, and incubated or not with exS100B. Stable expression of the RAGE receptor induces the S-nitrosylation of proteins involved in axon guidance, metabolism of proteins, and major pathway of rRNA processing in the nucleolus and cytosol terms, but the denitrosylation of proteins involved in IL-12 signaling and glycolysis. Stimulation with low and high exS100B-disturbed S-nitrosylation of proteins in CHOWT cells, mainly engaged in such biological reaction pathways as rRNA processing, cellular responses to stress, metabolism of RNA, or translation.

### 3.8. Validation of the LC-MS Identifications of Differentially S-Nitrosylated Proteins by Western Blot Analysis

We performed a western blot detection of four randomly selected proteins, enriched from the whole protein fractions, using the Biotin Switch Technique to validate our mass spectrometry identifications of S-NO proteins. We used protein-specific antibodies. We detected the levels of four proteins chosen that (1) have different functions and localization in cells, (2) the SNOSID/MS experiments showed their different S-nitrosylation patterns, depending on the concentration of exS100B and the RAGE receptor’s expression. The proteins selected for Western blot analysis were (1) Glyceraldehyde 3-phosphate dehydrogenase (GAPDH)-a cytoplasmic and nuclear protein and a key enzyme in glycolysis, with many non-metabolic roles; (2) Cathepsin D (CTSD)–a lysosomal aspartyl protease formed by disulfide connected heavy and light chains produced from a single protein precursor. S-nitrosylation of CTSD inhibits its processing to the active protease; (3) Superoxide dismutase [Cu-Zn], SOD1–an enzyme responsible for destroying free superoxide radicals in the body, and (4) heterogeneous nuclear ribonucleoprotein K (hnRPN K)–a gene expression regulator, both at the transcriptional and post-transcriptional levels and modulator of the inflammatory response by controlling the expression of TNFα, IL-1β, cycloox-ygenase-2, and iNOS; hnRPN K’s S-nitrosylation results in a thiol-reversible DNA binding inhibition.

**Figure 6:**
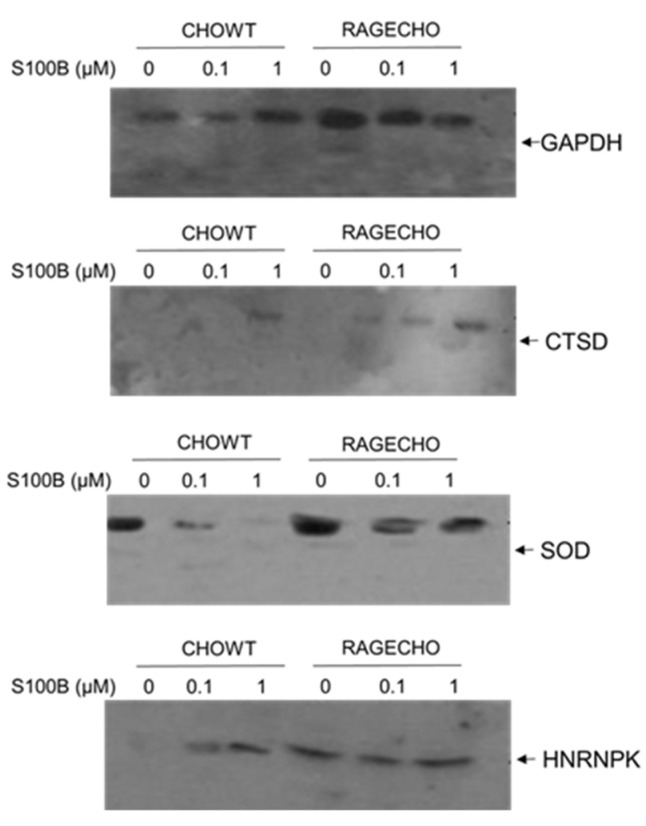
Protein-specific western blot validation of differentially S-nitrosylated in CHOWT and RAGECHO cells after treatment with 0.1 µM and 1.0 µM concentrations of S100B for glyceraldehyde dehydrogenase (GAPDH), cathepsin D (CTSD), superoxide dismutase 1 (SOD), and heterogeneous nuclear ribonucleoprotein K (HNRNPK).

Figure 6 shows the S-nitrosylation levels of four proteins, enriched using BST from identical amounts of protein fractions, extracted from cells under all studied experimental conditions; western blot analysis results are consistent with the MS-SNOSID study. As in MS results, S-nitrosylated GAPDH is present under all conditions. WB, however, provides additional information on different modulation of S-nitrosylation levels in the western blot analysis result, highest in untreated RAGE-expressing cells. Cathepsin D S-nitrosylation increases if cells are incubated with high amounts of exS100B, but otherwise is almost undetectable in CHOWT cells, and at a low level in RAGECHO cells. An opposite situation occurs for SOD-1 nitrosylation; it decreases in cells treated with exS100B.

The WB analysis supports the MS-based results, though performed for only four out of 101 identified proteins, and our hypothesis that variable levels of different sets of S-nitrosylated proteins are unique features of the exS100B or RAGE receptor-dependent cellular regulation. As shown in Figure 6, the WB-detected SNO level’s pattern is different for each protein.

Thus, it becomes clear that more detailed and quantitative S-nitrosome studies should provide additional crucial insight into the SNO-based regulatory mechanism beyond this work’s range.

## 4. Discussion

The data obtained in this study indicate that CHO cells well mimic some of the exS100B-related effects, observed in different human cell types, in particular, the concentration-dependent exS100B-induced expression of iNOS protein. Additionally, micromolar, but not nanomolar exS100B, induced the expression of the hamster RAGE receptor in CHOWT. A similar dependence effect was observed previously for astrocytes, where exS100B induced the expression of RAGE receptor through activation of Sp1 and NF-κB transcription factors [55]. Such observations convinced us to use the CHO cells as a testing model that extracellular S100B and RAGE receptors, together or separately, may induce an intracellular signaling response, based on the S-nitrosylation of protein cysteines.

Our work provides lists of specific proteins that are (1) elements of the S-nitrosome in untreated wild-type CHO cells, or in CHO cells with stable expression of human RAGE receptor, (2) the intracellular SNO targets in CHOWT or CHORAGE cells treated with nano- or micromolar exS100B. We highlighted proteins and S-nitrosylation sites from the lists common to all biological setups and specific to particular conditions. As mentioned in the results, we expect our protein and SNO site lists to be incomplete. The concentration of some SNO targets in cells could be too low for SNOSID/MS-based detection. Furthermore, the stabilities of specific S-nitrosylation sites in proteins differ significantly [56]. This could be a yet unidentified cellular response, induced to achieve beneficial or harmful effects.

Typically, for -omics studies, we used Gene Ontology annotations to find terms most impacted by SNO, in every studied condition (Figure 5). However, we note that this type of analysis has its limitations: (1) the fold enrichment analysis neglects the relationships between GO terms [57], (2) for proteins with multiple unrelated functions, modifications may not regulate them equally. Because of these reasons to draw more biologically relevant conclusions, we opted to base our discussion on specific proteins described by the GO terms identified during the fold enrichment analysis.

Protein S-nitrosylation is a regulator of cellular homeostasis, in many ways [53]. Usually, a subset of cellular proteins is constitutively S-nitrosylated. It may serve cells as storage molecules for reactive nitrogen oxide (NO) [58,59]. S-nitrosylation of catalytic site cysteines regulates enzymes, by either preventing their overactivation or inducing activity. The SNO group also protects some cysteine thiols from random, irreversible derivatization by other oxidative molecules [60]. This mechanism may be partly responsible for safeguarding astrocytes by exS100B from H_2_O_2_-induced cell death [61].

Most proteins modified by SNO in unstimulated CHOWT cells are enzymes involved in energy metabolism, including glycolysis, Krebs cycle, and respiratory chain. Many of them are multicysteine proteins, in which specific cysteines have already been detected in the S-nitrosomes of other cell types and tissues.

Under the six experimental conditions in this work, the modes of S-nitrosylation changes were not identical for individual proteins. For example, S-nitrosylation of Cys150, Cys154, and Cys245 in GAPDH; Cys293 in lactate dehydrogenase; Cys137 in cytoplasmic malate dehydrogenase, and Cys89, Cys93, Cys212, and Cys285 of mitochondrial malate dehydrogenase were stable under all conditions. The Cys45 in aldose reductase, Cys18, Cys19, and Cys369 from PHGDH and Cys293 of lactate dehydrogenase are S-nitrosylated in all CHOWT (independently of exS100B presence), but not in RAGECHO cells. Treatment of CHOWT cells with 0.1 µM exS100B is the only condition under which the Cys253 of PHGDH and Cys244 of triosephosphate isomerase are S-nitrosylated, and the Cys89 thiol in succinate dehydrogenase is free. In all other experiments, this residue was the only S-nitrosylated (out of 18 cysteines) in the dehydrogenase’s sequence.

The relevance of SNO at specific sites for protein function is yet unknown for the vast majority of S-nitrosation targets. A recent study used metabolomic profiling to elucidate the effect of SNO on the enzymatic activity of six enzymes, detected as S-nitrosylated in mouse liver proteins, under physiological conditions [61]. It turned out that, in only four enzymes, including the PHGDH caught in this work, S-nitrosylation influenced the levels of enzyme substrates and products. It was not the case for triose-phosphate isomerase and aldolase A. Thus, the modification must regulate other, yet unknown, protein functions. The best-known case for SNO regulation, different from the canonical enzymatic activity, is GAPDH. S-nitrosylated GAPDH binds Siah protein. Then, it is transported to the nucleus and functions there as a transnitrosylase for nuclear proteins [62]. The secretion or release of S100B relates to cell metabolism regulation, in processes involved in neurite extension [63], cancer [64], muscle regeneration [65], and restricted energy in sarcopenia [1,66]. In the same S100B-regulated processes, a key role is the regulation of local protein synthesis. Herein, we detected SNO sites in multiple ribosomal proteins in CHOWT and RAGECHO; however, in the latter case, more SNO proteins were associated with the organelle attached and not the cytosolic ribosomes. The two kinds of ribosomes produce different protein types in the cell [67]. A protein in which SNO influences cytoplasmic, but not canonical, nuclear functions, is the proliferating cell nuclear antigen (PCNA) [45]. Cys-162 of PCNA is SNO in all experiments in our study, but Cys81 only in CHOWT cells independent of exS100B. Cys81 nitrosylation inhibits an interaction of PCNA and caspase-9, which blocks caspase-9 activation in apoptosis [51]. The lysosomal aspartate protease cathepsin D is another vital participant in apoptotic processes. A low concentration of exS100B, induces in CHOWT cells, the SNO of cathepsins Cys329. This modification inhibits the efficient processing of the active protease [52,68].

**Figure 7:**
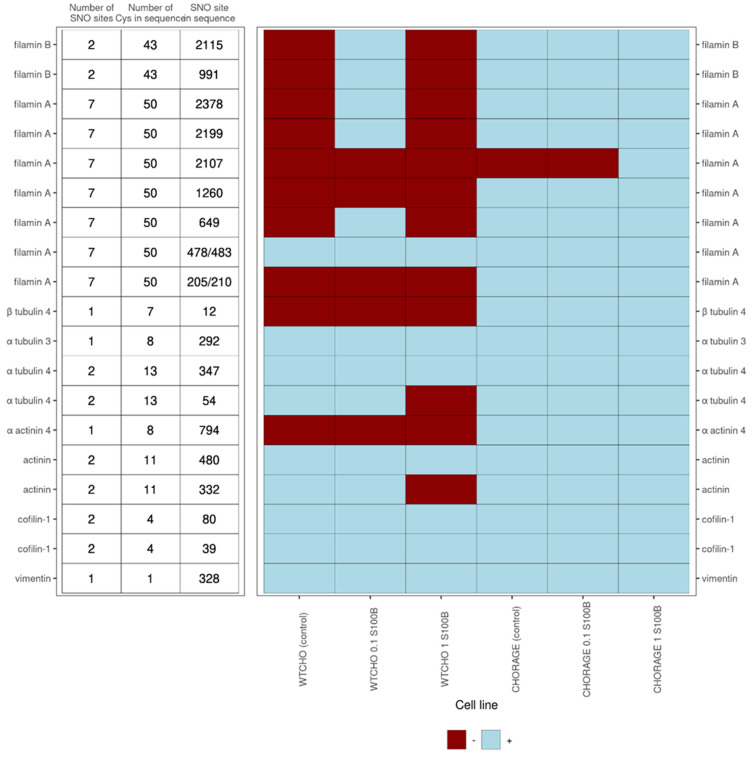
ExS100B or RAGE-regulated S-nitrosylation sites in cytoskeletal proteins for control CHOWT and RAGECHO cell cultures and the cells treated with 0.1 μM or 1.0 μM extracellular S100B. Indicated are the number of cysteine residues in a protein, the number of SNO sites detected for a protein under any experimental condition studied and the SNO site’s specific position in a protein. The presence of an SNO at a particular cysteine site is marked with burgundy color, the absence with blue.

S100B interactions with intracellular proteins are regulators of cell proliferation, differentiation, shape, and the dynamics of microtubules and type III intermediate filaments [1,12]. This work shows that exS100B may tune these processes through S-nitrosylation of cytoskeletal proteins and their interactors [66,69]. Figure 7 presents the detected-in-this-work complicated correlation of S-nitrosylation patterns in microfilament proteins: alpha-actinin, cofilin 1, filamin A and B; tubulin alpha and beta from microtubules, and the intermediate filament protein vimentin. Except for vimentin, which is a single cysteine protein, all others are multi cysteine proteins that are selectively S-nitrosylated at specific sites. So far, the functions described for these S-nitrosylations are that (1) VEGF-induced S-nitrosylation of cofilin 1 activates F-actin’s depolymerization and inhibits G-actin’s polymerization ability [70], (2) in vimentin, S-nitrosylation and S-glutathionylation of Cys328 regulate the tetramer/filament formation equilibrium—a process crucial for cell shape and motility [71].

Data obtained in this study also lead to interesting conclusions regarding the consequences of RAGE receptors’ expression at the cell surface. In WT CHO cells, stimulated with high levels of S100B, the induced levels of RAGE and iNOS proteins are similar to their levels in unstimulated RAGECHO cells. However, there are striking differences in the S-nitrosylated proteins. Many more targets are S-nitrosylated in the latter cells, and their number weakly changes after any exS100B stimulation. Furthermore, there is no enrichment in the SNO protein set from RAGECHO cells in glycolytic enzymes and proteins related to the interleukin-2-induced signaling pathway. Rather, nitrosylated are proteins interacting with cadherin, structural components of the ribosome, associated with rRNA processing. Based on these observations, it is tempting to propose that the different effects of the same RAGE levels in cells, either induced by a ligand or in the presence of the same amount of ligand, but previously stably transfected, may be a reflection of the different role of RAGE receptors in lungs [19,20,72]. RAGE expression is high in the lung under normal physiological conditions, unlike in other tissues [73]. There are hypotheses that RAGE may have unique roles, restricted to the pulmonary environment. In the lungs, RAGE modulates cell spreading, adhesion to ECM components, proliferation, and migration [74,75]. Disturbed-attenuated basal expression level results in impaired functioning of RAGE, giving rise to pathological states, including cancer and fibrosis. Another possibility is that the RAGECHO cells represent the receptors’ activated state, in which RAGE is a constitutive oligomer, even without ligands, just as when expressed on the surface of HEK293 cells [75].

Previously, we determined that S100A1 and S100B protein may be S-nitrosylated inside cells. The modification tuned the Ca^2+^ and Zn^2+^ binding properties and influenced the interaction of S100B with the p53 protein [76,77]. This study expands the knowledge on S-nitrosylation roles in the biology of the S100B protein. It shows that, in model mammalian cells, the network of intracellular protein post-translational S-nitrosylations senses the presence and level of extracellular S100B and the stable or S100B-induced expression of the RAGE receptor.

Presented data are consistent with the accepted role for protein S-nitrosylation, as a regulatory PTM [45,58,69,78]. The alterations of cellular S-nitrosome detected in this work include new target proteins, denitrosylations, and hierarchical nitrosylations of the same target.

In conclusion, our analysis revealed that tuning the intracellular protein S-nitrosome may represent a yet unknown cellular response, induced to achieve the beneficial or harmful effects of exS100B or the Receptor for Advanced Glycation End products. It is a matter for future studies, to analyze the S-NO-related pathways, in the context of other S100B interactors on the cell surface, and other RAGE receptor ligands (i.e., S100 proteins, Alzheimer peptide, advanced glycation end products, and others [22]) and in cells and conditions better representing the various physiological ex S100B and RAGE environment.

We propose that studying intracellular S-nitrosylation, along with other PTMs, is necessary to understand better signaling pathways, in systems relevant to the development of tailored therapies for S100B and RAGE receptor-related disease.

## Figures and Tables

**Table 1:** Proteins, specifically S-nitrosylated, depending on the concentration of exS100B used to treat CHOWT or RAGECHO cells.

Occurrence of Differential S-Nitrosylated Proteins in CHOWT Cells	Gene Names	Protein Names
SNO present only in control cells	-	-
SNO absent only in control cells	ACIN1 PPP2R1B HNRNPK ERP44	Apoptotic chromatin condensation inducer in the nucleus, Protein Ppp2r1b, Heterogeneous nuclear ribonucleoprotein K, Endoplasmic reticulum resident protein 44
SNO present only with 0.1 μM S100B	CTSD FLNB ABCE1	Cathepsin D, Filamin B, ATP-binding cassette sub-family E member 1
SNO absent only with 0.1 μM S100B	SDHA PCBP2	Succinate dehydrogenase [ubiquinone] flavoprotein subunit, mitochondrial Poly(rC)-binding protein 2
SNO present only with 1 μM S100B	SNRPD3 HNRNPL PDIA3	Small nuclear ribonucleoprotein Sm D3, Heterogeneous nuclear ribonucleoprotein L, Protein disulfide-isomerase A3
SNO absent only with 1 μM S100B	UBE2D2 SOD1 SSRP1 RPS3A ACAT3 ATP2A2	Ubiquitin-conjugating enzyme E2 D2, Superoxide dismutase [Cu-Zn], FACT complex subunit SSRP1, 40S ribosomal protein S3a, Acetyl-Coenzyme A acetyltransferase 3, Sarcoplasmic/endoplasmic reticulum calcium ATPase 2

**Table 2:** S-nitrosylation sites differentiate the CHOWT S-nitrosomes depending on the concentration of exS100B used to treat cells.

Occurrence of Differential S-Nitrosylated Sites in CHOWT Cells	Gene Names	Protein Names
SNO present only in control cells	ANXA11	Annexin A11
SNO absent only in control cells	TCP1 DPYSL2 PGK1 VCP RPS8 ATP1A1	T-complex protein 1, subunit alpha, Dihydropyrimidinase-related protein 2, Phosphoglycerate kinase 1, Transitional endoplasmic reticulum ATPase, 40S ribosomal protein S8, Sodium/potassium-transporting ATPase subunit alpha-1
SNO present only with 0.1 μM S100B	RPL12 ENO1 IPO5 PLEC FLNA PRDX4 PHGDH PRMT1 HSPH1 GOT2	60S ribosomal protein L12, Alpha-enolase, Importin-5, Plectin, Filamin-A, Peroxiredoxin-4, D-3-phosphoglycerate dehydrogenase, Protein arginine n-methyltransferase 1, Heat shock protein 105 kDa, Aspartate aminotransferase, mitochondrial
SNO absent only with 0.1 μM S100B	PABPC1 HSPH1 TPI1d1	Polyadenylate-binding protein 1, Heat shock protein 105 kDa, Triose-phosphate isomerase
SNO present only with 1 μM S100B	LDHA SUCLG1 GNB2	L-lactate dehydrogenase A chain, Succinate-CoA ligase [ADP/GDP-forming] subunit alpha, mitochondrial, Guanine nucleotide-binding protein G(I)/G(S)/G(T) subunit beta-2,
SNO absent only with 1 μM S100B	ACTN1 PRDX5 TUBA4A PABPC1	Alpha-actinin-1, Peroxiredoxin-5, mitochondrial, Tubulin alpha-4A chain, Polyadenylate-binding protein 1

**Table 3:** S-nitrosylated protein sites differentiate S-nitrosomes of RAGECHO cells depending on the concentration of exS100B used to treat cells.

Occurrence of Differential S-Nitrosylated Sites in RAGECHO Cells	Gene Names	Protein Names
Present only in control cells	-	-
Absent only in control cells	-	-
Present only with 0.1 μM S100B	-	-
Absent only with 0.1 μM S100B	PGAM1 PDIA3 RCC2 NOP56 GNB2	Phosphoglycerate mutase 1, Protein disulfide-isomerase A3, Protein RCC2, Nucleolar protein 56, Guanine nucleotide-binding protein G(I)/G(S)/G(T) subunit beta-2
Present only with 1 μM S100B	FLNA	Filamin-A
Absent only with 1 μM S100B	_	_

## Data Availability

Not applicable.

## Supplementary Materials

The following supporting information can be downloaded at: https://www.mdpi.com/article/10.3390/biom12050613/s1, Table S1: Peptide ions detected in the fraction of S-nitrosylated peptides selectively enriched by SNOSID; Table S2: List of proteins differentiating the S-nitrosome of CHOWT cells depending on the concentration of exS100B used to treat cells. Table S3: List of S-nitrosylation sites differentiating the CHOWT S-nitrosomes depending on the concentration of exS100B used to treat cells. Table S4: S-nitrosylated protein sites differentiating the S-nitrosomes of RAGECHO cells depending on the concentration of the exS100B used to treat cells.
