# Supplementary material for: Intracellular Protein S-Nitrosylation—A Cells Response to Extracellular S100B and RAGE Receptor
Source: Biomolecules. 2022 Apr 20;12(5):613. doi: 10.3390/biom12050613 (PMC9138530; doi:10.3390/biom12050613)
Supplement: Supplementary file 1 [file biomolecules-12-00613-s001.zip › biomolecules-1477814-SI.pdf]

Table S1. Peptide ions detected in the fraction of S-nitrosylated peptides selectively enriched by SNOSID; **+** and **–** describe the presence or absence of a peptide ion in CHOWT and RAGE CHO cell lines treated or not with exS100B.

Tryptic peptide sequence (charge)	SPROT name	CHO WT CTR	CHO WT 0.1 µM S100B	CHO WT 1 µM S100B	RAGE CHO CTR	RAGE CHO 0.1 µM S100B	RAGE CHO 1 µM S100B
VSVVDLTCR (+2)	P16858	-	-	-	+	+	+
VPTPNVSVVDLTCR (+2)	P16858	+	+	+	+	+	+
IVSNASCTTNCLAPLAK (+2)	P16858	+	+	+	+	+	+
ENECHFYAGGQVYPGEVSR (+3)	O08807	+	+	-	+	+	+
SINTEVVACSVDSQFTHLAWINTPR (+3)	O08807	+	+	+	+	+	+
HGEVCPAGWKPGSETIIPDPAGK (+3)	O08807	-	+	+	+	+	+
STLTDSLVCK (+2)	P58252	+	+	+	+	+	+
RCLYASVLTAQPR (+2)	P58252	+	+	+	+	+	+
ETVSEESNVLCLSK (+2)	P58252	+	-	+	+	+	+
KIWCFGPDGTGPNILTDITK (+3)	P58252	-	-	-	+	+	+
YVEPIEDVPCGNIVGLVGVDQFLVK (+3)	P58252	+	+	+	+	+	+
CEFQDAYVLLSEK (+2)	P63038	+	+	+	+	+	+
AAVEEGIVLGGGCALLR (+2)	P63038	+	+	+	+	+	+
IAVAAQNCYK (+2)	P17751	-	-	-	+	+	+
CLGELICTLNAAK (+2)	P17751	+	+	+	+	+	+
VSHALAEGLGVIACIGEK (+3)	P17751	-	-	-	+	+	+
AQCPIVER (+2)	P97461	+	+	+	+	+	+
TIAECLADELINAAK (+2)	P97461	+	+	+	+	+	+
RVNQAIWLLCTGAR (+3)	P97461	-	-	-	+	+	+
GIFPVLCK (+2)	XP574742	+	+	+	+	+	+
CCSGAIIVLTK (+2)	XP574742	+	+	+	+	+	+
NTGIICTIGPASR (+2)	XP574742	+	+	+	+	+	+
CDENILWLDYK (+2)	XP574742	+	+	+	+	+	+
SFDDSGNGYCR (+2)	P19096	-	-	-	+	+	+
VYATILNAGTNTDGCK (+2)	P19096	-	-	-	+	+	+
EACPELDYFVAFSSVSCGR (+2)	P19096	-	-	-	+	+	+
GCDVVVIPAGVPR (+2)	P08249	+	+	+	+	+	+
TIIPLISQCTPK (+2)	P08249	+	+	+	+	+	+
GYLGPEQLPDCLK (+2)	P08249	+	+	+	+	+	+
ETECTYFSTPLLLGK (+2)	P08249	+	+	+	+	+	+
FGANAILGVSLAVCK (+2)	P17182	-	-	-	+	+	+
VNQIGSVTESLQACK (+2)	P17182	+	+	+	+	+	+
SCNCLLLK (+2)	P17182	+	+	+	+	+	+
FCLDNGAK (+2)	P09411	-	+	+	+	+	+
GCITIIGGGDTATCCAK (+2)	P09411	+	+	+	+	+	+
DCVGPEVENACANPAAGTVILLENLR (+3)	P09411	+	+	+	+	+	+
ECADLWPR (+2)	P62830	+	+	+	+	+	+
ISLGLPVGAVINCADNTGAK (+2)	P62830	+	+	+	+	+	+
LVILANNCPALR (+2)	P62889	+	+	+	+	+	+
VCTLAIIDPGDSDIIR (+2)	P62889	+	+	+	+	+	+
CTGGEVGATSALAPK (+2)	P35979	-	+	+	+	+	+
EILGTAQSVGCNVDGR (+2)	P35979	-	+	-	+	+	+
GPQVCGELK (+2)	Q61484	-	-	-	+	+	+

GPSVDVEVPDVDLECPDAK (+2)		+	+	+	+	+	+
DSNNLCLHFNPR (+2)	P48538	+	+	+	+	+	+
FNAHGDANTIVCNSK (+2)		+	+	+	+	+	+
NCIVLIDSTPYR (+2)	P62242	-	-	+	+	+	+
LLACIASRPGQCGR (+3)		+	+	+	+	+	+
LDVGNFSWGSECCTR (+2)		-	+	+	+	+	+
TLIQNCGASTIR (+2)	P80318	-	-	-	+	+	+
IPGGIIEDSCVLR (+2)		+	+	+	+	+	+
WSSLACNIALDAVK (+2)		+	+	+	+	+	+
GLGTDEDSLIEIICSR (+2)		+	+	+	+	+	+
QVQSLTCEVDALK (+2)	P20152	+	+	+	+	+	+
SIQFVDWCPTGFK (+2)	P68368	+	+	+	+	+	+
TIGGGDDSFTTFFCETGAGK (+2)		+	+	+	+	+	+
INISEGNCPER (+2)	P60335	+	+	+	+	+	+
LVVPATQCGSLIGK (+2)		+	+	+	+	+	+
VGLTNYAAAYCTGLLLAR (+2)	P47962	-	-	-	+	+	+
GAGTDEACLIEILASR (+2)	P97384	+	+	-	+	+	+
GFGTDEQAIIDCLGSR (+2)		+	+	+	+	+	+
IECDDKGDGSCDVR (+2)	Q8BTM8	-	-	-	+	+	+
SPYTVTVGQACNPAACR (+2)		+	+	+	+	+	+
VTYCPTEPGNYIINIK (+2)		-	-	-	-	-	+
THEAEIVEGENHTYCIR (+3)		-	+	-	+	+	+
YWPQEAGEYAVHVLCNSEDIR (+3)		-	+	-	+	+	+
LQVEPAVDTSGVQCYGPGIEGQGVFR (+3)		-	+	-	+	+	+
VHSPSGALEECYVTEIDQDKYAVR (+4)		-	+	-	+	+	+
ALGALVDSCAPGLCPDWDSW-DASKPVNNAR (+3)		-	-	-	+	+	+
ALAGCDFLTISPK (+2)	Q93092	+	+	+	+	+	+
ALDLDSSCK (+2)	Q60864	-	-	-	+	+	+
ALSAGNIDDALQCYSEAIK (+2)		+	+	+	+	+	+
GYEVIYLTEPVDEYCIQALPEFDGK (+3)		-	-	-	+	+	+
GLCAIAQAESLR (+2)	P62908	-	-	-	+	+	+
GLELIASENFCSR (+2)	Q99K87	+	+	+	+	+	+
VLELVSITANKNTCPGDR (+3)		+	+	+	+	+	+
YYGGAEVVDEIELLCQR (+3)		+	+	+	+	+	+
ENFSCLTR (+2)	P14152	+	+	+	+	+	+
VIVVGNPANTNCLTASK (+2)		+	+	+	+	+	+
VLISDSLDPCCR (+2)	Q61753	-	-	-	+	+	+
NAGTCLSPAVIVGLLR (+2)		+	+	+	+	+	+
ALVDHENVISCPHLGASTK (+3)		+	+	+	+	+	+
ALQSGQCAGAALDVFTEEPPRDR (+3)		-	+	-	+	+	+
IYHPNINSNGSICLDILR (+3)	P62838	+	+	-	+	+	+
YVDIAIPCNNK (+2)	P14206	+	+	+	+	+	+
ADHQPLTEASYVNLPTIALCNTDSPLR (+3)		+	+	+	+	+	+
ACVVHGSDLK (+2)	Q8VDN2	-	-	-	+	+	+
LIIVEGCQR (+2)		+	+	+	+	+	+
NIAFFSTNCVEGTAR (+2)		-	+	+	+	+	+
NLEAVETLGSTSTICSDK (+2)		-	-	-	+	+	+
DAGYEFDICFTSVQK (+2)	Q9DBJ1	-	-	-	+	-	+

YADLTEDQLPSCESLKDTIAR (+3)		+	+	+	+	+	+
DLTTAGAVTQCYR (+2)	P62717	+	+	+	+	+	+
MLPDKDCR (+2)	P18760	+	+	+	+	+	+
AVLFCLSEDKK (+2)		+	+	+	+	+	+
HELQANCYEEVKDR (+3)		-	-	-	+	+	+
TIQFVDWCPTGFK (+2)	P05214	+	+	+	+	+	+
CVESFSDYPPLGR (+2)	P10126	-	-	-	+	+	+
KDGSASGTTLLEALDCILPPTRPTDKPLR (+4)		-	-	-	+	+	+
GVLFYGPPGCGK (+2)	P46462 Q01853	+	+	+	+	+	+
LGDVISIQPCPDVK (+2)	P46462	-	+	+	+	+	+
EAVCIVLSDDTCSDEK (+2)		-	-	-	+	+	+
QAAPCVLFFDELDSIAK (+2)		+	+	+	+	+	+
VVETDPSPYCIVAPDTVIHCEGEPIK (+3)		-	-	-	+	+	+
LVVPASQCGSLIGK (+2)	Q61990	+	-	+	+	+	+
ALANSLACQGK (+2)	P05064	+	+	+	+	+	+
CPLLKPWALTFSYGR (+3)		-	-	-	+	+	+
YASICQQNGIVPIVEPEILPDGDHDLKR (+4)		+	+	+	+	+	+
LVICPDEGFYK (+2)	P61082	+	+	+	+	+	+
TCDISFSDPDDLLNFK (+2)		+	+	+	+	+	+
GLYDGPVCEVSVTPK (+2)	O08553	+	+	+	+	+	+
SITIANQTNCPLYVTK (+2)		+	+	+	+	+	+
VIGSGCNLDSAR (+2)	P06151	+	+	+	+	+	+
VICQGFTGK (+2)	Q9WUM5	-	-	+	+	+	+
LIGPNCPGIINPGECK (+2)		+	+	+	+	+	+
HEQNIDCGGGYVK (+2)	P14211	+	+	+	+	+	+
CKDDEFTHLYTLIVRPDNTYEVK (+4)		+	+	-	+	+	+
KITISDCGQL (+2)	P17742	+	+	+	+	+	+
IIPGFMCQGGDFTR (+2)		+	+	+	+	+	+
HTGPGILSMANAGPNTNGSQFFICTAK +OXIDATION(M) (+3)		-	+	+	+	+	+
GGCEAIVDTGTSLLVGPVDEVK (+2)	P18242	-	+	-	+	+	+
SNIWVAGDAACFYDIK (+2)	Q9Z0X1	+	+	+	+	+	+
GFGFVCFSSPEEATK (+2)	P29341	+	-	+	+	+	+
ALYDTFSAFGNILSCK (+2)		+	+	-	+	+	+
AAFGLSEAGFNTACLTK (+2)	Q8K2B3	+	-	+	+	+	+
AGAIAPCEVTVPAQNTGLGPEK (+2)	P14869	+	+	+	+	+	+
TAVCDIPPR (+2)	Q9D6F9	-	-	-	+	+	+
EIVHLQAGQCGNQIGAK (+3)		-	-	-	+	+	+
VGTQTFCSR (+2)	Q3U7R1	+	+	+	+	+	+
ATYSTNCPVWEEAFR (+2)		+	+	+	+	+	+
DFTPVCTTELGR (+2)	AAC67553 O08709	+	+	+	+	+	+
TFVSGACDASIK (+2)	P62880	-	-	-	+	+	+
ACGDSTLTQITAGLDPVGR (+3)		+	+	+	+	+	+
SVSAFAPICNPVLCPWGK (+2)	XP_484341	+	+	+	+	+	+
LSIQCYLSALDR (+2)	Q8JZK9	-	-	-	+	+	+
PGSLPLNAEACWPK (+2)		-	-	-	+	+	+

CITDPQTGLCLLPLK (+2)	P30427 Q9QXS1	+	+	+	+	+	+
FLEGTSCIAGVFVDATK (+2)	P30427	+	+	+	+	+	+
TLLQGSGCLAGIYLEDSK (+2)		-	+	+	+	+	+
FICQQEHEK (+2)	Q60446/ Q61699	-	+	-	+	+	+
NAVEECVYEFR (+2)	Q60446	-	+	-	+	+	+
GCALQCAILSPAFK (+2)		+	-	+	+	+	+
LCFSTAQHAS (+2)	Q8R081	-	-	+	+	+	+
VFNVFCLYGNVEK (+2)		-	-	-	+	+	+
VVPCLVAPVAGR (+2)	Q80X90	-	+	-	+	+	+
APSVATVGSICDLNLK (+2)		-	+	-	+	+	+
GAGTDEGCLIEILASR (+2)	P97429	+	+	+	+	+	+
SSGEIVYCGKVFEK (+2)	D3YVT7	-	-	-	+	+	+
VDCDQHSDIAQR (+2)	Q9D1Q6	-	+	+	+	+	+
TPADCPVIAIDSFR (+2)		-	-	-	+	+	+
IDCFSEVPTSVFGEK (+2)	Q9D6Z1	-	-	-	+	+	+
LVAFCPFSSSQVALENANAV-SEGVVHEDLR (+3)		-	-	-	+	-	+
LLLECAR (+2)	Q8BKC5	+	+	+	+	+	+
TIECISLIGLAVGK (+2)		-	-	-	+	+	+
VQAHAAAALINFTEDCPK (+3)		-	+	-	+	+	+
ICDQWDNLGALTQK (+2)	Q7TPR4	+	+	+	+	+	+
CQLEINFNTLQTK (+2)		+	+	+	+	+	+
IACLDFSLQK (+2)	P11984	+	+	+	+	+	+
ICDDELILIK (+2)		-	+	+	+	+	+
QLVTNACLIK (+2)	Q9JIF0	-	-	-	+	+	+
GQLCELSCSTDYR (+2)		-	-	-	+	+	+
VIGIECSSISDYAVK (+2)		-	-	-	+	+	+
VEDLTFTSPFCLQVK (+2)		+	+	+	+	+	+
AVQDLCGWR (+2)	Q8BK67	-	-	-	+	+	+
IEYDCELVPR (+2)		-	-	-	+	+	+
DVACGANHTLVLDSQK (+2)		-	-	-	+	+	+
GNLYSFGCPEYGQLGHNSDGK (+3)		-	-	-	+	+	+
LICCDILDVLDK (+2)	A2A5N2	-	-	-	+	+	+
LFCVGFTK (+2)	P97351	+	+	-	+	+	+
ACQSIYPLHDVFVR (+3)		+	+	-	+	+	+
VPFLVLECPNLK (+2)	AAK69656 Q78XF5	+	+	+	+	+	+
VAPEEVSEVIFGHVLTAGCGQNPTR (+3)	Q80X81	+	+	+	+	+	+
VVNEINIEDLCLTK (+2)		+	+	+	+	+	+
YLECSALTQR (+2)	P60764	+	+	+	+	+	+
HHCPNTPIILVGTK (+3)		-	-	-	+	+	+
VGEATETALTCLVEK (+2)	O55143	+	+	-	+	+	+
SLPSVETLGCTSVICSDK (+2)		-	+	-	+	+	+
VGAFTVVCK (+2)	P05202	+	+	+	+	+	+
TCGFDFSGALEDISK (+2)		+	+	+	+	+	+
NLDKEYLPIGGLAEFCK (+3)		+	+	+	+	+	+
STCLYGQLPK (+2)	P46425	+	+	+	+	+	+
ADVIQATGDAICIFR (+2)	Q08943	+	+	-	+	+	+
GVLFGVPGAFTPGCSK (+2)	P99029	+	+	-	+	+	+

ALNVEPDGTGLTCSLAPNILSQL (+2)		+	+	+	+	+	+
ACLISLGYDVENDR (+2)	P57780	-	-	-	+	+	+
VVCEDFLQDVAASTK (+2)	Q9R152<br>P11103	-	-	-	+	+	+
LQPIALSCVLNIGACK (+2)	Q9CR16	-	-	-	+	+	+
VLLDAPCSGTGVISK (+2)	Q922K7	+	+	+	+	+	+
GSDFDCELR (+2)	P61979	-	+	+	+	+	+
LFQECCPHSTDR (+3)		-	-	-	+	+	+
VQTDPPSVPICDLYPNGVFPK (+2)	O08663	+	+	+	+	+	+
GDFCIQVGR (+2)	P15532	+	+	+	+	+	+
SCAQNWIYE (+2)		+	+	+	+	+	+
VFVLPCIQQIQR (+2)	O08917	+	+	+	+	+	+
TSACGLFSVCYPR (+2)	Q8BIX1	-	+	+	+	+	+
LNIISNLDCVNEVIGIR (+2)		-	-	-	+	+	+
TLGECGFTSQTARPQAPATVGLAFR (+3)	P62869	-	-	-	+	+	+
AAPCIYWLPLTESQIVQK (+2)	Q9JIX8	+	+	+	+	+	+
DPPEEEIPFCTLK (+2)		-	+	+	+	+	+
HIDCAQVYQNEK (+2)	P45376	-	-	-	+	+	+
VDCTANTNTCNK (+2)	P27773	-	-	+	+	+	+
FIQESIFGLCPH (+2)	Q91Z81	-	-	+	+	+	+
HNLCGETEEER (+2)	O35660	+	+	+	+	+	+
TQAIVCQQLDLTHLK (+3)	P61222	-	-	-	+	+	+
CPFGALSIVNLPSNLEK (+2)		-	+	-	+	+	+
CAGNEDIITLR (+2)	P17918	-	-	-	+	+	+
DLSHIGDAVVISCAK (+2)		+	+	+	+	+	+
AVCVLKGDGPVQGTIHFEQK (+3)	P08228	+	+	-	+	+	+
NLFEDQNTLTSICEK (+2)	Q9ERK4	-	-	-	+	+	+
VLHEAEGHIVTCETNTGEVYR (+3)	P62320	-	-	+	+	+	+
GLYGIKDDVFLSVPCVLGQNGISDVVK (+3)	P06151	-	-	+	+	+	+

Table S2. List of proteins differentiating the S-nitrosome of CHOWT cells depending on the concentration of exS100B used to treat cells.

	SPROT name	Protein name	Gene Ontology Molecular Function	Gene Ontology Biological Process
**Presence of SNO only in CHOWT CTR**	-	-	-	-
**Lack of SNO only in CHOWT CTR**	ACIN1	Apoptotic chromatin condensation inducer in the nucleus	nucleic acid binding	apoptotic chromosome condensation, apoptotic process, negative regulation of mRNA splicing, via spliceosome
	PPP2R1B	Protein Ppp2r1b	DNA binding	-
	HNRNPK	Heterogeneous nuclear ribonucleoprotein K	actinin binding, ATPase binding, DNA binding, C-rich single-stranded DNA binding, DNA-binding transcription activator activity, RNA polymerase II-specific, double-stranded DNA binding, heat shock protein binding, identical protein binding, RNA binding	cellular response to forskolin, cellular response to insulin stimulus, cerebral cortex development, modulation of chemical synaptic transmission, mRNA processing, negative regulation of apoptotic process, negative regulation of branching
	ERP44	Endoplasmic reticulum resident protein 44	protein disulfide isomerase activity	cell redox homeostasis, glycoprotein metabolic process, protein folding, response to endoplasmic reticulum stress, response to unfolded protein
**Presence of SNO only in CHOWT 0.1 µM S100B**	CTSD	Cathepsin D	aspartic-type endopeptidase activity, aspartic-type peptidase activity, endopeptidase activity, hydrolase activity, peptidase activity, peptide binding	autophagosome assembly, lipoprotein catabolic process, positive regulation of apoptotic process, positive regulation of cysteine-type, proteolysis, regulation of establishment of protein localization
	FLNB	Filamin B	actin binding, identical protein binding	actin cytoskeleton organization, cellular response to interferon-gamma, epithelial cell morphogenesis, keratinocyte development, skeletal muscle tissue development
	ABCE1	ATP-binding cassette sub-family E member 1	ATPase activity, ATP binding, endoribonuclease inhibitor activity, iron ion binding, ribosomal small subunit binding	negative regulation of endoribonuclease activity, ribosomal subunit export from nucleus, translational initiation, translational termination
**Lack of SNO only in CHOWT with 0.1 µM S100B**	SDHA	Succinate dehydrogenase [ubiquinone] flavoprotein subunit, mitochondrial	electron transfer activity, flavin adenine dinucleotide binding, oxidoreductase activity, succinate dehydro-	mitochondrial electron transport, succinate to ubiquinone, nervous system development, oxidation-reduction process, respiratory electron

			genase (ubiquinone) activity, succinate dehydrogenase activity	transport chain, succinate metabolic process, tricarboxylic acid cycle
	PCBP2	Poly(rC)-binding protein 2	C-rich single-stranded DNA binding, DNA binding, enzyme binding, RNA binding, ubiquitin protein ligase binding	defense response to virus, innate immune response, IRES-dependent viral translational initiation, negative regulation of defense response to virus, proteasome-mediated ubiquitin-dependent protein catabolic process, viral RNA genome replication
**Presence of SNO only in CHOWT 1 μM S100B**	SNRPD3	Small nuclear ribonucleoprotein Sm D3	enzyme binding, histone pre-mRNA DCP binding, RNA binding, telomerase RNA binding	mRNA splicing, via spliceosome, protein methylation, spliceosomal snRNP assembly
	HNRNPL	Heterogeneous nuclear ribonucleoprotein L	mRNA 3'-UTR binding, mRNA binding, mRNA CDS binding , pre-mRNA intronic binding , RNA binding , transcription regulatory region, DNA binding	cellular response to amino acid starvation, circadian rhythm, RNA processing, negative regulation of mRNA splicing,
	PDIA3	Protein disulfide-isomerase A3	identical protein binding, MHC class I protein binding, peptidase activity, peptide disulfide oxidoreductase activity	cell redox homeostasis, cellular response to interleukin-7, cellular response to vitamin D, positive regulation of extrinsic apoptotic
**Lack of SNO only in CHOWT 1 μM S100B**	UBE2D2	Ubiquitin-conjugating enzyme E2 D2	ATP binding, ubiquitin conjugating enzyme activity, ubiquitin protein ligase binding, ubiquitin-protein transferase activity	protein autoubiquitination, protein K48-linked ubiquitination, protein polyubiquitination, protein ubiquitination, ubiquitin-dependent protein catabolic process
	SOD1	Superoxide dismutase [Cu-Zn]	chaperone binding, copper ion binding, enzyme binding, identical protein binding, protein phosphatase 2B binding, Rac GTPase binding, superoxide dismutase activity, zinc ion binding	activation of MAPK activity, aging, anterograde axonal transport, auditory receptor cell stereocilium organization, cell aging, cellular iron ion homeostasis, cellular response to ATP, cellular response to cadmium ion
	SSRP1	FACT complex subunit SSRP1	chromatin binding, DNA binding, histone binding, nucleosome binding	DNA repair, DNA replication, regulation of chromatin organization
	RPS3A	40S ribosomal protein S3a	mRNA 5'-UTR binding, mRNA binding, structural constituent of ribosome, translation initiation factor binding	cell differentiation, negative regulation of apoptotic process, positive regulation of fibroblast proliferation, positive regulation of translation, translation

	ACAT3	Acetyl-Coenzyme A acetyltransferase 3	acetyl-CoA C-acetyltransferase activity, acetyl-CoA C-acyltransferase activity	fatty acid beta-oxidation
	ATP2A2	Sarcoplasmic/endoplasmic reticulum calcium ATPase 2	ATP binding, calcium ion binding, calcium transmembrane transporter activity, phosphorylative mechanism, calcium-transporting ATPase activity	calcium ion import into sarcoplasmic reticulum, calcium ion transmembrane transport, calcium ion transport, calcium ion transport from cytosol to endoplasmic reticulum, cardiac muscle hypertrophy in response to stress

Table S3 List of S-nitrosylation sites differentiating the CHOWT S-nitrosomes depending on the concentration of exS100B used to treat cells

	SPROT name	Protein name	Gene Ontology Molecular Function	Gene Ontology Biological Process
**Presence of SNO only in CHOWT CTR**	ANXA11	Annexin A11	calcium-dependent phospholipid binding, calcium-dependent protein binding, calcium ion binding, phosphatidylethanolamine binding, S100 protein binding	cytokinetic process, phagocytosis, response to calcium ion
**Lack of SNO only in CHOWT CTR**	TCP1	T-complex protein 1 subunit alpha	ATP binding, ubiquitin protein ligase binding, unfolded protein binding	binding of sperm to zona pellucida, positive regulation of establishment of protein localization to telomere, positive regulation of telomerase activity, protein folding, protein stabilization, regulation of macrophage apoptotic process, scaRNA localization to Cajal body, toxin transport, translocation of peptides or proteins into host cell cytoplasm
	DPYSL2	Dihydropyrimidinase-related protein 2	hydrolase activity, acting on carbon-nitrogen (but not peptide) bonds, in cyclic amides, identical protein binding, microtubule binding, protein kinase binding	axon guidance, cytoskeleton organization, endocytosis, olfactory bulb development, positive regulation of glutamate secretion, regulation of axon extension, regulation of neuron differentiation, regulation of neuron projection development, response to amphetamine, response to cocaine, spinal cord development, synaptic vesicle transport
	PGK1	Phosphoglycerate kinase 1	ADP binding, ATP binding, phosphoglycerate kinase activity, protein-disulfide reductase activity	carbohydrate metabolic process, cellular response to hypoxia, epithelial cell differentiation, gluconeogenesis, glycolytic process, negative regulation of angiogenesis, phosphorylation, plasminogen activation, positive regulation of oxidative phosphorylation
	VCP	Transitional endoplasmic reticulum ATPase	ADP binding, ATPase activity, ATP binding, BAT3 complex binding, deubiquitinase activator activity, identical protein binding, K48-linked polyubiquitin modification-dependent protein binding, lipid binding, MHC class I	activation of cysteine-type endopeptidase activity involved in apoptotic process, aggresome assembly, ATP metabolic process, autophagosome maturation, autophagy, cellular response to arsenite ion, cellular response to DNA damage stimulus, cellular

			protein binding, polyubiquitin modification-dependent protein binding, protein-containing complex binding, protein domain specific binding, protein phosphatase binding, signaling receptor binding, ubiquitin-like protein ligase binding, ubiquitin protein ligase binding, ubiquitin-specific protease binding	response to heat, double-strand break repair, endoplasmic reticulum stress-induced pre-emptive quality control, endoplasmic reticulum to Golgi vesicle-mediated transport, endosome to lysosome transport via multivesicular body sorting pathway, ERAD pathway, ER-associated misfolded protein catabolic process, flavin adenine dinucleotide catabolic process, macroautophagy
	RPS8	40S ribosomal protein S8	structural constituent of ribosome	maturation of SSU-rRNA from tricistronic rRNA transcript (SSU-rRNA, 5.8S rRNA, LSU-rRNA), translation
	ATP1A1	Sodium/potassium-transporting ATPase subunit alpha-1	ADP binding, ankyrin binding, ATP binding, chaperone binding, phosphatase activity, phosphatidylinositol 3-kinase binding, potassium ion binding	cellular potassium ion homeostasis, cellular response to mechanical stimulus, cellular response to steroid hormone stimulus, cellular sodium ion homeostasis, membrane hyperpolarization, membrane repolarization
**Presence of SNO only in CHOWT 0.1 μM S100B**	RPL12	60S ribosomal protein L12	large ribosomal subunit rRNA binding, rRNA binding, structural constituent of ribosome	ribosomal large subunit assembly, translation
	ENO1	Alpha-enolase	DNA-binding transcription repressor activity, RNA polymerase II-specific, enzyme binding, GTPase binding, heat shock protein binding, identical protein binding, magnesium ion binding	canonical glycolysis, cellular response to interleukin-7, glycolytic process, in utero embryonic development, positive regulation of binding
	IPO5	Importin-5	nuclear import signal receptor activity, nuclear localization sequence binding, Ran GTPase binding	cellular response to amino acid stimulus, NLS-bearing protein import into nucleus, positive regulation of protein import into nucleus, protein import into nucleus, ribosomal protein import into nucleus
	PLEC	Plectin	actin binding, ankyrin binding, cytoskeletal protein binding, protein N-terminus binding, structural constituent of cytoskeleton, structural constituent of muscle, structural molecule activity	epithelial cell differentiation, female pregnancy, hemidesmosome assembly, intermediate filament cytoskeleton organization, response to nutrient, wound healing
	FLNA	Filamin-A	actin binding, actin filament binding, Fc-gamma receptor	actin crosslink formation, actin cytoskeleton organization, actin

			I complex binding, G protein-coupled receptor binding, GTPase binding, ion channel binding, kinase binding, Rac GTPase binding, Ral GTPase binding, Rho GTPase binding, SMAD binding, small GTPase binding, transcription factor binding	cytoskeleton reorganization, adenylate cyclase-inhibiting dopamine receptor signaling pathway, angiogenesis, blood vessel remodeling, cell-cell junction organization, cilium assembly, cytoplasmic sequestering of protein, early endosome to late endosome transport, epithelial to mesenchymal transition, establishment of protein localization
	PRDX4	Peroxiredoxin-4	thioredoxin peroxidase activity	cell redox homeostasis, extracellular matrix organization, male gonad development, negative regulation of male germ cell proliferation, oxidation-reduction process, peptidyl-proline hydroxylation to 4-hydroxy-L-proline, protein maturation by protein folding, reactive oxygen species metabolic process, response to oxidative stress, spermatogenesis
	PHGDH	D-3-phosphoglycerate dehydrogenase	NAD binding, phosphoglycerate dehydrogenase activity	cellular amino acid metabolic process, G1 to G0 transition, gamma-aminobutyric acid metabolic process, glial cell development, glutamine metabolic process, glycine metabolic process, L-serine biosynthetic process, L-serine metabolic process, neural tube development, neurogenesis
	PRMT1	Protein arginine N-methyltransferase 1	[cytochrome c]-arginine N-methyltransferase activity, enzyme binding, histone-arginine N-methyltransferase activity, histone methyltransferase activity, identical protein binding, methyl-CpG binding, mitogen-activated protein kinase p38 binding	histone H4-R3 methylation, histone methylation, in utero embryonic development, negative regulation of megakaryocyte differentiation, neuron projection development, peptidyl-arginine methylation, peptidyl-arginine methylation, to asymmetrical-dimethyl arginine, peptidyl-arginine omega-N-methylation, positive regulation of cell population proliferation
	HSPH1	Heat shock protein 105 kDa	adenyl-nucleotide exchange factor activity, alpha-tubulin binding, ATP binding	chaperone cofactor-dependent protein refolding, negative regulation of apoptotic signaling pathway, negative regulation of establishment of protein localization to mitochondrion, negative regulation of intrinsic apoptotic signaling pathway in response to

				hydrogen peroxide, negative regulation of neuron apoptotic process, negative regulation of p38MAPK cascade
	GOT2	Aspartate aminotransferase, mitochondrial	amino acid binding, carboxylic acid binding, enzyme binding, identical protein binding, kynurenine-oxoglutarate transaminase activity, L-aspartate:2-oxoglutarate aminotransferase activity, phospholipid binding, pyridoxal phosphate binding	2-oxoglutarate metabolic process, aspartate biosynthetic process, aspartate catabolic process, aspartate metabolic process, cellular amino acid metabolic process, dicarboxylic acid metabolic process, fatty acid transport, female pregnancy
**Lack of SNO only in CHOWT 0.1 μM S100B**	PABPC1	Polyadenylate-binding protein 1	mRNA 3'-UTR binding, mRNA binding, poly(A) binding, poly(U) RNA binding, protein C-terminus binding, RNA binding	gene silencing by RNA, mRNA processing, negative regulation of nuclear-transcribed mRNA catabolic process, nonsense-mediated decay, nuclear-transcribed mRNA catabolic process, nonsense-mediated decay, positive regulation of nuclear-transcribed mRNA catabolic process, deadenylation-dependent decay, positive regulation of nuclear-transcribed mRNA poly(A) tail shortening, positive regulation of viral genome replication, RNA splicing
	HSPH1	Heat shock protein 105 kDa	adenyl-nucleotide exchange factor activity, alpha-tubulin binding, ATP binding	chaperone cofactor-dependent protein refolding, negative regulation of apoptotic signaling pathway, negative regulation of establishment of protein localization to mitochondrion, negative regulation of intrinsic apoptotic signaling pathway in response to hydrogen peroxide, negative regulation of neuron apoptotic process, negative regulation of p38MAPK cascade, positive regulation of MHC class I biosynthetic process, positive regulation of NK T cell activation, positive regulation of protein tyrosine kinase activity, positive regulation of transcription by RNA polymerase II, regulation of microtubule cytoskeleton organization, response to unfolded protein
	TPI1d1	Triose-phosphate isomerase	isomerase activity, methylglyoxal synthase activity,	Gluconeogenesis, glucose metabolic process, glyceraldehyde-3-

			protein homodimerization activity, triose-phosphate isomerase activity, ubiquitin protein ligase binding	phosphate biosynthetic process, glyceraldehyde-3-phosphate metabolic process, glycerol catabolic process l, glycolytic process, methylglyoxal biosynthetic process, multicellular organism development
**Presence of SNO only in CHOWT 1 μM S100B**	LDHA	L-lactate dehydrogenase A chain	cadherin binding, identical protein binding, kinase binding, L-lactate dehydrogenase activity, NAD binding	glycolytic process, lactate metabolic process, NAD metabolic process, positive regulation of apoptotic process, response to estrogen, response to glucose, response to hydrogen peroxide, response to hypoxia, response to nutrient
	SUCLG1	Succinate--CoA ligase [ADP/GDP-forming] subunit alpha, mitochondrial	cofactor binding, GDP binding, nucleoside diphosphate kinase activity, protein-containing complex binding	nucleoside triphosphate biosynthetic process, succinate metabolic process, succinyl-CoA metabolic process, tricarboxylic acid cycle
	GNB2	Guanine nucleotide-binding protein G(I)/G(S)/G(T) subunit beta-2	calcium channel regulator activity, GTPase activity, GTPase binding protein-containing complex binding	G protein-coupled receptor signaling pathway
**Lack of SNO only in CHOWT 1 uM S100B**	ACTN1	Alpha-actinin-1	actin filament binding, calcium ion binding, double-stranded RNA binding, integrin binding, ion channel binding, nuclear receptor transcription coactivator activity, protein domain specific binding, protein homodimerization activity, structural constituent of postsynapse, vinculin binding	actin crosslink formation, actin filament bundle assembly, actin filament network formation, actin filament organization, cortical cytoskeleton organization, focal adhesion assembly, negative regulation of cellular component movement, platelet formation, platelet morphogenesis
	PRDX5	Peroxiredoxin-5, mitochondrial	antioxidant activity, cysteine-type endopeptidase inhibitor activity involved in apoptotic process, peroxidase activity, peroxiredoxin activity, peroxynitrite reductase activity, RNA polymerase III regulatory region DNA binding, signaling receptor binding, thioredoxin peroxidase activity	cell redox homeostasis, cellular response to oxidative stress, cellular response to reactive oxygen species, hydrogen peroxide catabolic processNADPH oxidation, negative regulation of apoptotic process, negative regulation of oxidoreductase activity, negative regulation of transcription by RNA polymerase III, positive regulation of collagen biosynthetic process, reactive nitrogen species metabolic process, regulation of apoptosis involved in

				tissue homeostasis, response to oxidative stress
	TUBA4A	Tubulin alpha-4A chain	enzyme binding, GTPase activity, GTP binding, protein kinase binding, structural constituent of cytoskeleton	microtubule-based process, microtubule cytoskeleton organization, mitotic cell cycle
	PABPC1	Polyadenylate-binding protein 1	mRNA 3'-UTR binding, mRNA binding, poly(A) binding, poly(U) RNA binding, protein C-terminus binding, RNA binding	gene silencing by RNA, mRNA processing, negative regulation of nuclear-transcribed mRNA catabolic process, nonsense-mediated decay, nuclear-transcribed mRNA catabolic process, nonsense-mediated decay, positive regulation of nuclear-transcribed mRNA catabolic process, deadenylation-dependent decay, positive regulation of nuclear-transcribed mRNA poly(A) tail shortening, positive regulation of viral genome replication, RNA splicing

Table S4 S-nitrosylated protein sites differentiating the S-nitrosomes of RAGECHO cells depending on the concentration of the exS100B used to treat cells.

	Gene name	Protein name	Gene Ontology Molecular Function	Gene Ontology Biological Process
**Present only in RAGECHO CTR**	-	-	-	-
**Lack only in RAGECHO CTR**	-	-	-	-
**Present only in RAGE CHO 0.1 μM S100B**	-	-	-	-
**Lack only in RAGECHO 0.1 μM S100B**	PGAM1	Phosphoglycerate mutase 1	bisphosphoglycerate mutase activity, hydrolase activity, phosphoglycerate mutase activity, protein kinase binding	canonical glycolysis, gluconeogenesis, glycolytic process, neutrophil degranulation, regulation of glycolytic process, regulation of pentose-phosphate shunt, respiratory burst
	PDIA3	Protein disulfide-isomerase A3	identical protein binding, MHC class I protein binding, peptidase activity, peptide disulfide oxidoreductase activity, protein disulfide isomerase activity, protein-disulfide reductase (glutathione) activity	cell redox homeostasis, cellular response to interleukin-7, cellular response to vitamin D, positive regulation of extrinsic apoptotic signaling pathway, positive regulation of protein folding, protein folding, response to endoplasmic reticulum stress
	RCC2	Protein RCC2	guanyl-nucleotide exchange factor activity, microtubule binding, protein domain specific binding, protein kinase binding, Rac GTPase binding, small GTPase binding	activation of GTPase activity, cell cycle, cell division, chromosome passenger complex localization to kinetochore, establishment of protein localization, focal adhesion assembly, integrin-mediated signaling pathway, negative regulation of focal adhesion assembly, negative regulation of GTPase activity, negative regulation of substrate adhesion-dependent cell spreading, positive regulation of attachment of spindle microtubules to kinetochore, positive regulation of G2/M transition of mitotic cell cycle, regulation of cell migration, regulation of fibroblast migration, regulation of ruffle assembly

	NOP56	Nucleolar protein 56	histone methyltransferase binding, snoRNA binding	ribosome biogenesis
	GNB2	Guanine nucleotide-binding protein G(I)/G(S)/G(T) subunit beta-2	calcium channel regulator activity, GTPase activity, GTPase binding	G protein-coupled receptor signaling pathway
**Present only in RAGECHO 1μM S100B**	FLNA	Filamin-A	actin binding, actin filament binding, Fc-gamma receptor I complex binding, G protein-coupled receptor binding, GTPase binding, ion channel binding, kinase binding, mu-type opioid receptor binding, potassium channel regulator activity, protein-containing complex binding, protein homodimerization activity, protein kinase C binding, Rac GTPase binding, Ral GTPase binding, Rho GTPase binding, SMAD binding, small GTPase binding, transcription factor binding	actin crosslink formation, actin cytoskeleton organization, actin cytoskeleton reorganization, adenylate cyclase-inhibiting dopamine receptor signaling pathway, angiogenesis, blood vessel remodeling, cell-cell junction organization, cilium assembly, cytoplasmic sequestering of protein, early endosome to late endosome transport, epithelial to mesenchymal transition, establishment of protein localization, establishment of Sertoli cell barrier, formation of radial glial scaffolds, heart morphogenesis, mitotic spindle assembly, mRNA transcription by RNA polymerase II, negative regulation of apoptotic process, negative regulation of DNA-binding transcription factor activity, negative regulation of neuron projection development, negative regulation of protein catabolic process, negative regulation of transcription by RNA polymerase I
**Lack only in RAGECHO 1μM S100B**	–	–	–	–
